# Entropy–Entropy Compensation between the Protein, Ligand, and Solvent Degrees of Freedom Fine-Tunes Affinity in Ligand Binding to Galectin-3C

**DOI:** 10.1021/jacsau.0c00094

**Published:** 2021-04-01

**Authors:** Johan Wallerstein, Vilhelm Ekberg, Majda Misini Ignjatović, Rohit Kumar, Octav Caldararu, Kristoffer Peterson, Sven Wernersson, Ulrika Brath, Hakon Leffler, Esko Oksanen, Derek T. Logan, Ulf J. Nilsson, Ulf Ryde, Mikael Akke

**Affiliations:** † Biophysical Chemistry, Center for Molecular Protein Science, Department of Chemistry, Lund University, 221 00 Lund, Sweden; ∇ Theoretical Chemistry, Department of Chemistry, Lund University, 221 00 Lund, Sweden; § Biochemistry and Structural Biology, Center for Molecular Protein Science, Department of Chemistry, Lund University, 221 00 Lund, Sweden; ∥ Centre for Analysis and Synthesis, Department of Chemistry, Lund University, 221 00 Lund, Sweden; ⊥ The Swedish NMR Center, University of Gothenburg, 405 30 Gothenburg, Sweden; # Microbiology, Immunology, and Glycobiology, Department of Experimental Medicine, Lund University, 221 00 Lund, Sweden; ○ European Spallation Source ESS ERIC, 225 92 Lund, Sweden

**Keywords:** Drug design, Molecular recognition, Ligand binding specificity, Conformational entropy, Solvation entropy, Thermodynamics

## Abstract

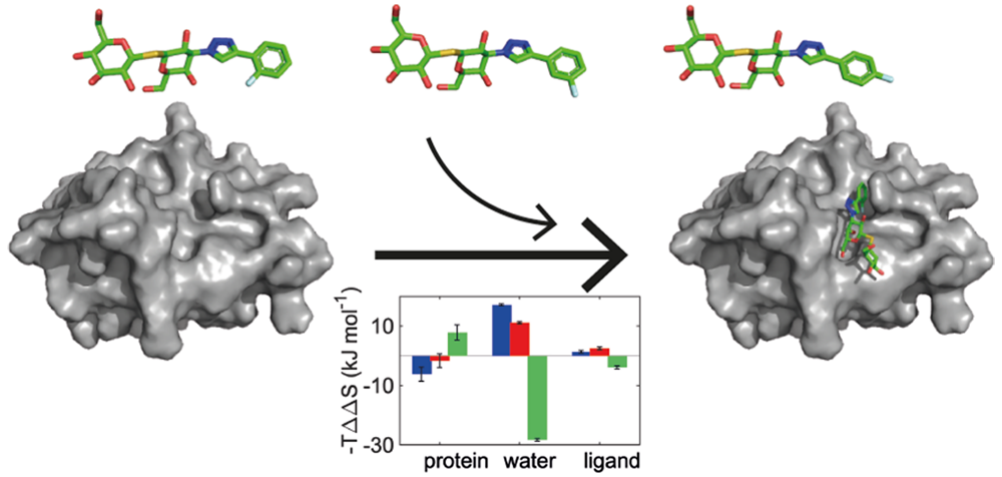

Molecular recognition is fundamental to biological signaling. A central question is how individual interactions between molecular moieties affect the thermodynamics of ligand binding to proteins and how these effects might propagate beyond the immediate neighborhood of the binding site. Here, we investigate this question by introducing minor changes in ligand structure and characterizing the effects of these on ligand affinity to the carbohydrate recognition domain of galectin-3, using a combination of isothermal titration calorimetry, X-ray crystallography, NMR relaxation, and computational approaches including molecular dynamics (MD) simulations and grid inhomogeneous solvation theory (GIST). We studied a congeneric series of ligands with a fluorophenyl-triazole moiety, where the fluorine substituent varies between the *ortho*, *meta*, and *para* positions (denoted O, M, and P). The M and P ligands have similar affinities, whereas the O ligand has 3-fold lower affinity, reflecting differences in binding enthalpy and entropy. The results reveal surprising differences in conformational and solvation entropy among the three complexes. NMR backbone order parameters show that the O-bound protein has reduced conformational entropy compared to the M and P complexes. By contrast, the bound ligand is more flexible in the O complex, as determined by ^19^F NMR relaxation, ensemble-refined X-ray diffraction data, and MD simulations. Furthermore, GIST calculations indicate that the O*-*bound complex has less unfavorable solvation entropy compared to the other two complexes. Thus, the results indicate compensatory effects from ligand conformational entropy and water entropy, on the one hand, and protein conformational entropy, on the other hand. Taken together, these different contributions amount to entropy–entropy compensation among the system components involved in ligand binding to a target protein.

## Introduction

Binding of low-molecular-weight ligands to proteins is fundamental to a large number of signaling pathways in biology and also of central interest in drug design aiming to interfere with such pathways for medicinal purposes. Rational structure-based design of effectors by computational approaches has advanced immensely in recent decades yet remains an extremely challenging endeavor. The main challenge involves the fact that the free energy of binding is manifested as a small difference between large numbers originating from a vast number of interactions between the protein, ligand, other solutes, and solvent molecules. The interaction energies depend sharply on interatomic distances and orientations, which vary in a time-dependent manner with the conformational dynamics. Furthermore, changes in conformational fluctuations in proteins upon ligand binding can give rise to significant entropic contributions to affinity.^1−8^ Ligand binding may also affect the water network in and around binding sites, causing appreciable effects on the thermodynamics of solvation.^8−13^

We have studied the carbohydrate recognition domain of galectin-3 (residues 113–250; denoted galectin-3C) to address specifically the roles of conformational entropy^4,5,8^ and solvation in ligand binding.^8,13^ Galectin-3 is an interesting model system in this regard, because its binding site is relatively solvent-accessible, being located in a shallow groove across one of the two β-sheets. Several water molecules form an integral part of the ligand environment by forming bridging hydrogen bonds between the ligand and protein.^13,14^ Galectin-3 is a member of the galectin family of mammalian lectins, which is defined by a conserved sequence motif that confers affinity for β-galactoside-containing glycans.^15,16^ Galectins play important roles in cell growth, cell differentiation, cell cycle regulation, signaling, and apoptosis, making them targets for medicinal chemistry programs to treat inflammation and cancer,^17,18^ with several cases reported for galectin-3.^19−21^ Recently, it has also been suggested that galectin-3 plays a role in the pathology of Alzheimer’s disease.^22^

Here, we investigate the effects of minute changes in ligand structure on the binding enthalpy and entropy, broken down into separate contributions from various interactions or degrees of freedom. We report a comparative analysis of galectin-3C in complex with three congeneric fluorophenyltriazolyl-thiogalactosides that differ by the position of the fluorine substituent, which is placed in the *ortho*, *meta*, or *para* position; henceforth, the ligands are denoted O, M, and P. The advantage of using ligands with minimal structural differences is that differences in binding thermodynamics are dominated by the properties of the ligand–protein complexes, while the chemical potential of the free ligands contributes relatively less in the comparative analysis. On the other hand, the minor structural differences between the ligands in this series lead us to expect limited differences in the structures of the complexes and in their binding thermodynamics. Thus, this study focuses on the thermodynamic effects of minute changes in protein–ligand structure and aims at characterizing the response of the entire system, including the solvent. In particular, we address how the various entropic contributions to binding might vary between slightly different protein–ligand complexes.

We used a combination of experimental and computational approaches, including isothermal titration calorimetry (ITC), competitive fluorescence polarization, X-ray crystallography, NMR relaxation, and molecular dynamics (MD) simulations followed by conformational entropy and grid inhomogeneous solvation theory (GIST) calculations. Our results show that minor differences between protein–ligand complexes in their overall binding thermodynamics might encompass greater differences among individual contributions, including a case of entropy–entropy compensation between the protein, ligand, and solvent degrees of freedom.

## Materials and Methods

### Ligand Synthesis

The synthesis of ligands M and P was performed as described for O.^23^ 1,3-Dipolar cycloaddition of 3-fluoro- and 4-fluorophenylacetylene to 3′-azido-3′-deoxy-β-d-galactopyranosyl-1-thio-β-d-glucopyranoside under Cu(I) catalysis afforded ligands M and P, respectively. Procedures and physical data for M and P are described in the Supporting Information.

### Protein Expression and Purification

Galectin-3C was expressed and purified following published protocols^4,5^ to produce ^15^N-, ^15^N/^13^C-, and ^15^N/^13^C/^2^H-labeled protein as well as unlabeled protein. The protein stock solution was 16 mg/mL in buffer, consisting of 10 mM Na_2_HPO_4_, 1.8 mM KH_2_PO_4_, 140 mM NaCl, 2.7 mM KCl, pH 7.3, 2 mM ethylenediaminetetraacetic acid (EDTA), 4 mM tris(2-carboxyethyl)phosphine hydrochloride (TCEP), and 150 mM lactose. The protein stock solution was stored at 278 K.

### Isothermal Titration Calorimetry

^15^N-labeled galectin-3C samples were prepared by extensive dialysis (Slide-A-Lyzer MINI Dialysis ThermoScientific) against 5 mM 4-(2-hydroxyethyl)-1-piperazinethanesulfonic acid (HEPES) buffer to remove all lactose, followed by centrifugation at 14 000 rpm for 5 min to remove any aggregates. The protein concentration was determined by UV absorption at 280 nm, as described before.^4^ The ligands are water-soluble and do not require addition of DMSO. The three ligands were dissolved into 7–10 mM stock solutions using the same HEPES buffer as used in the protein samples and sonicated immediately prior to experiments. ITC experiments were performed on a MicroCal PEAQ–ITC instrument (Malvern Panalytical) at pH 7.3 and a temperature of 301.1 K by titrating the protein at a concentration of 100 ± 4 μM into the cell containing the ligand at a concentration of 1000 ± 10 μM. Two replicate experiments were performed for each complex. The time between injections was set to 4 min. Peak integration was done using NITPIC,^24^ with individual error bars assigned to each injection. A single-site binding model was fitted simultaneously to the two titration curves to yield the binding enthalpy (Δ*H°*), fraction of binding-competent protein (*n*), and dissociation constant (*K*_d_), using ITCsy.^25^ The heat released or absorbed during the *i*th injection is given by^26^
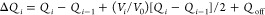
where *V*_*i*_ is the volume of the *i*th injection, *V*_0_ is the cell volume, *Q*_off_ is an offset parameter accounting for the heat of mixing, and *Q*_*i*_ is the heat following the *i*th injection
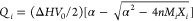
where α = *nM*_*i*_ + *X*_*i*_ + *K*_d_ and *M*_*i*_ and *X*_*i*_ are the total concentrations of protein and ligand, respectively, in the cell at any given point of the titration.

The free energy and entropy of binding were determined via the equations Δ*G°* = *RT* ln(*K*_d_) and −*T*Δ*S°* = Δ*G°* – Δ*H°*. The statistical uncertainty is greater for −*T*Δ*S°* than for Δ*G°* or Δ*H°*, since it is derived from two parameters. ITCsy/SEDPHAT produces asymmetric error estimates, but in the present case, the errors are nearly symmetric, and we report the errors as the average of upper and lower errors.

### X-ray Crystallography

Small crystals of lactose-bound galectin-3C were grown with the hanging drop method in NeXtal plates (Qiagen) and with the following reservoir condition: 20% (w/v) PEG 4000, Tris-HCl pH 7.5, 0.4 M NaSCN, 10 mM β-mercaptoethanol. The drop volume was 2 μL, and the protein solution/reservoir ratio was 1:1. Small crystals were then moved to drops containing the same reservoir with the addition of 10 mM of the ligand (O, M, or P), from a 100 mM stock solution in PBS buffer. Soaking lasted for 12–15 h. Before data collection, crystals were placed for a couple of seconds in a drop containing 1 volume of 100% PEG 400 and 3 volumes of crystallization solution to prevent ice formation during cryo-cooling. Data were collected on EMBL beamline P13 at PETRA-III, DESY, Hamburg.^27^ For each complex, 3600 diffraction images were collected with 0.1° rotation and 0.01 s exposure time. All data were integrated using XDS.^28^

Scaling and merging were done with Aimless.^29^ Cross-validation was based on 10% of the reflections. Molecular replacement was done using Phaser in the Phenix suite version 1.14,^30,31^ using as template the lactose–galectin-3C structure^13^ with lactose and water molecules removed. Ligands and their crystallographic restraints were generated through phenix.eLBOW.^32^ Restrained refinement was then performed using phenix.refine^33^ using all remaining reflections. Manual rebuilding, including addition of water molecules, was done using Coot.^34^ Data collection and refinement statistics for analysis of X-ray crystallography data are found in Table S1.

### Ensemble Refinement of Crystal Structures

Ensemble refinement of the X-ray diffraction data was performed using the module phenix.ensemble_refinement^35^ in the Phenix 1.14 software suite. The X-ray crystal structures of the M, P, or O complexes were used as starting structures. The crystallographic water molecules were kept, and hydrogen atoms and missing atoms in the protein were added using the phenix.ready_set module. Ligand restraints and coordinates were the same as those used in the original refinement.

The collective dynamics of the protein was described using a TLS (translation–libration–screw) model with a single group, which included both the protein and the ligand atoms. The percentage of atoms included in the TLS fitting (*p*_TLS_) was optimized by testing five different values (0.5, 0.6, 0.7, 0.8, and 0.9) and choosing the one that yielded the lowest *R*_free_, which was *p*_TLS_ = 0.6 for all three protein–ligand complexes. An ensemble of structures was then generated by running MD simulations, in which the model was restrained by a time-averaged X-ray maximum-likelihood target function. Three X-ray weight-coupled temperature bath offsets were tested (2.0, 5.0, and 10.0 K), with the default value of 5 K giving the lowest *R*_free_. A 1.25 ps relaxation time of the time-averaged restraints was used, resulting in 25 ps long MD simulations, with structures stored every 0.05 ps. All structures generated by ensemble refinement were kept, resulting in 500 different structures in each ensemble. Atomic fluctuations were calculated using the *cpptraj* module of Amber after removal of the water molecules.^36^

### NMR Sample Preparation

Samples of ^15^N-labeled, ^15^N/^13^C-labeled, or ^15^N/^13^C/^2^H-labeled galectin-3C in complex with each of the M, P, and O ligands were prepared in the same buffer as used for ITC. Samples were prepared by titrating 5 μL of ligand stock solution in 6–9 steps to the NMR tubes containing a protein concentration of 0.8–0.9 mM and recording a ^15^N-heteronuclear single quantum correlation (HSQC) spectrum at each titration point. The resulting saturation levels were 98.0–98.5%, as calculated from the value of *K*_d_ determined by ITC measurements. In addition, ^15^N-labeled samples of each complex were obtained by pooling the cell contents from the two replicate ITC experiments, resulting in a galectin-3C concentration of 0.08 mM. ^15^N-HSQC spectra were acquired on these samples to confirm that the samples resulting from the ITC titrations are essentially identical to those used in the NMR relaxation experiments.

### NMR Resonance Assignments and Chemical Shift Perturbation Analysis

Backbone chemical shift assignments were based on HNCACB^37^ and CBCA(CO)NH)^38^ spectra and previous assignments for various galectin-3C complexes.^5^ The ligands were titrated into the NMR tubes in five to seven steps so as to follow the movement of individual resonances from the apo state to the ligand-bound state. Methyl groups were assigned using ^13^C-HSQC, CCH-TOCSY, and HCCH-TOCSY experiments^39,40^ and previous assignments.^5^ All spectra were processed using NMRPipe,^41^ employing a solvent filter, squared cosine apodization, linear prediction, and zero filling to twice the number of points in all dimensions. Resonance assignments were done using the CcpNmr program suite.^42^ Chemical shift perturbations were calculated as ([Δδ(^1^H)]^2^ + [0.16Δδ(^15^N)]^2^)^1/2^ for backbone amides and ([Δδ(^1^H)]^2^ + [0.25Δδ(^13^C)]^2^)^1/2^ for methyl-bearing side chains.

### NMR Relaxation Experiments and Data Analysis

Backbone amide ^15^N *R*_1_, *R*_2_, and {^1^H}–^15^N nuclear Overhauser effect (NOE) experiments were performed at static magnetic field strengths of 11.7, 14.1, and 18.8 T and a temperature of 301 K. Temperature calibration was done prior to each relaxation series experiment using a type-T copper-constantan thermocouple element with one electrode in an ice–water bath and the other in an NMR tube in water, positioned at the sample location inside the magnet. Experiments at 11.7 and 14.1 T were performed on Varian/Agilent VNMRS DirectDrive spectrometers, and experiments at 18.8 T were performed on a Bruker Avance III HD spectrometer. The spectral widths were 14–16 and 28–30 ppm for ^1^H and ^15^N, respectively, covered by 1024 and 128 points. Relaxation decays were recorded with 10 relaxation delays ranging between 0–1 s for *R*_1_ and 0–0.2 s for *R*_2_ with a 1.2 ms delay between refocusing pulses. The NOE experiments at 11.7, 14.1, and 18.8 T all used the same ^1^H saturation time of 7 s, and the recycle delay between experiments was 3, 7, and 3 s, respectively. The reference experiment (without ^1^H saturation) was acquired using a recycle delay of 10, 14, and 10 s at 11.7, 14.1, and 18.8 T, respectively. All spectra were processed as described above. Relaxation rates were extracted using PINT, which employs line-shape fitting to resolve overlapped peaks.^43^ Peak intensities were evaluated using a weighted sum of Lorentzian and Gaussian line shapes. Monoexponential functions were fitted to the *R*_1_ and *R*_2_ relaxation decays using a jackknife error estimation.^44^ NOEs were calculated as the ratio of the peak intensities in the saturated and reference experiments, and the standard deviations were determined by propagating the errors of intensities estimated from the baseplane noise. Error estimates are reported as 1 standard deviation (SD).

Methyl ^2^H relaxation experiments^45^ measuring *R*_1_(*D*_*Z*_), *R*(3*D_Z_*^2^ – 2), *R*_2_(*D*_+_), and *R*(*D*_+_*D*_*Z*_ + *D*_*Z*_*D*_+_) were recorded at 11.7 and 14.1 T. Spectral widths were 16 and 20 ppm for ^1^H and ^13^C, respectively, covered by 1024 points in the ^1^H dimension at both field strengths and by 70 and 84 points for ^13^C at 11.7 and 14.1 T, respectively. Relaxation decays were sampled by 9 points covering 0–0.1 s for *R*_1_(D_Z_) and *R*(3D_Z_^2^–2) and 0–20 ms for *R*_2_(D_+_) and *R*(D_+_D_Z_+D_Z_D_+_). The recycle delay was 1.8–2 s. Peak intensities were evaluated as described above.

^19^F relaxation *R*_2_ experiments were recorded at 470 and 659 MHz on Agilent/Varian VNMRS and Bruker spectrometers, respectively. The temperature was calibrated to 301 ± 0.1 K using a methanol reference sample. ^1^H decoupling during the relaxation period was achieved using a 180° pulse or WALTZ train. The experiment at 470 MHz utilized a spectral width of 4596 Hz, 3584 transients, relaxation delays of 0, 4, 8 (×2), 12, 16, 20, 28, and 40 ms, and a recycle delay of 4 s. The experiment at 659 MHz utilized a spectral width of 13 158 Hz, 512 transients, relaxation delays of 0, 2, 4 (×2), 6, 8 (×2), 12, 16, 20 (×2), 28, 32, and 40 ms, and a recycle delay of 5 s. Both experiments were run in an interleaved manner. All ^19^F spectra were processed using NMRPipe^41^ with zero filling and matched filter apodization. Peak intensities were evaluated using in-house MATLAB scripts. Errors in peak intensities were estimated from the baseline noise. Monoexponential functions were fitted to the *R*_2_ decays using least-squares optimization functions available in MATLAB, with parameter uncertainties estimated from 1000 Monte Carlo simulations.^46^

### Model-Free Analysis of NMR Relaxation Data

Backbone NH model-free parameters^47,48^ were fitted using the *relax* software package (v 4.0.2),^49−51^ with an NH bond length of 1.02 Å and a ^15^N chemical shift anisotropy of −172 ppm. Hydrogen atoms were added to the PDB structures using UCSF Chimera.^52^ The backbone optimization in *relax* was restricted to the four most basic models defined by the parameter sets: {*O*^2^}, {*O*^2^, τ_e_}, {*O*^2^, *R*_ex_}, or {*O*^2^, τ_e_, *R*_ex_}, where *O*^2^ denotes the generalized order parameter, τ_e_ denotes the effective correlation time for the internal motion, and *R*_ex_ denotes exchange contributions to *R*_2_; in addition, the correlation time for overall rotational diffusion τ_c_, was fitted as well as the diffusion tensor.^53^ The definitions of anisotropy (ζ) and rhombicity (η) of the diffusion tensor^54^ are ζ = 2*D*_*ZZ*_/(*D*_*XX*_ + *D*_*YY*_) and η = 1.5(*D*_*YY*_ – *D*_*XX*_)/[*D*_*ZZ*_ – 0.5(*D*_*XX*_ + *D*_*YY*_)], where {*D*_*XX*_, *D*_*YY*_, *D*_*ZZ*_} are the principal components of the molecular rotational diffusion tensor.

Side-chain methyl-axis model-free optimization was performed using in-house MATLAB routines employing the fmincon function to find the minimum of a constrained nonlinear multivariable function. Numerically stabilized model-free equations suggested previously were used.^51^ Three models were fitted using two {*O*^2^, τ_f_}, three {*O*^2^, τ_f_, τ_eff_}, or four {*O*_f_^2^, *O*_s_^2^, τ_f_, τ_eff_} parameters, where τ_f_ is associated with fast motions, τ_eff_ = (1/τ_c_ + 1/τ_s_)^−1^, and τ_s_ denotes the correlation time for slow internal motions on par with τ_c_.^55^ The global correlation time and diffusion tensor were fixed to values obtained from the ^15^N backbone model-free optimization in *relax*. Model selection was performed using an *F* test at the level α = 0.95 (*p* < 0.05).^56^ Error estimates, reported as 1 SD, were based on Monte Carlo simulations using 500 samples.^46^

### Comparison of Three Complexes

We report the comparisons of the three complexes as the difference between the parameter value of a certain property of complex A minus the average value for the other two complexes, B and C, i.e., Δ*P*(A) = *P*(A) – (*P*(B) + *P*(C))/2. The values of Δ*P* resulting from this intercomplex comparison are a factor of 1.5 greater than differences calculated relative to the average value of all three complexes. The sum of all three Δ*P*(i) is zero.

### Conformational Entropy Estimates from Order Parameters

The change in backbone conformational entropy, comparing two states, was estimated from the NMR order parameters using the relationship^1,57^
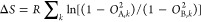
1where *R* is the gas constant and the sum runs over all residues *k*. Using the intercomplex comparison described above, the equation becomes
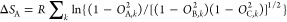
2

The conformational entropy change of the side-chain methyl axis was calculated as^57^
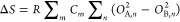
3where *C*_*m*_ is a parameter that depends on the residue type: *C*_*m*_ = 1.32 for Val and Thr, 3.1 for Ile and Leu, and 2.31 for Met.^57^ The sums run over all residues *n* of type *m*. Since eq 3 is linear, the intercomplex difference is calculated in a straightforward manner. The entropy for Ala side chains was calculated using eq 1.

### Radial Distribution of Conformational Entropy

The radial distribution of protein conformational entropy around the bound ligand was calculated by averaging the backbone entropy of residues located in 1–2 Å thick shells surrounding the ligand. The distance from the ligand was evaluated as the shortest distance between any ligand atom and any atom of a given residue in the corresponding X-ray crystal structure. The residues were sorted into shells with a thickness of 1 Å (2–9 Å) or 2 Å (9–27 Å); the thinner shells were used to improve the resolution of the data close to the ligand. The lowest entropy value for each complex was taken as the reference and set to 0. The cumulative average entropy values were also computed as a function of the distance from the ligand.

### Molecular Dynamics Simulations and Analysis

All MD simulations were run with the Amber software suite.^58^ The X-ray crystal structures of the three complexes were used as the starting points for MD simulations. All crystal-water molecules were kept in the simulations. Each galectin-3C complex was solvated in an octahedral box of water molecules extending at least 10 Å from the protein using the *tleap* module. The simulations were set up in the same way as in our previous studies of galectin-3C.^4,8,59,60^ All Glu and Asp residues were assumed to be negatively charged, and all Lys and Arg residues positively charged, whereas the other residues were neutral. The binding-site residue His158 was protonated on the ND1 atom, whereas the other three His residues were protonated on the NE2 atom, in accordance with the neutron structure and NMR measurements^14^ as well as previous extensive test calculations with MD.^61^ This resulted in a net charge of +4 for the protein. No counterions were used in the simulations.

The protein was described by the Amber ff14SB force field,^62^ water molecules with the TIP4P-Ewald model,^63^ whereas the ligands were treated with the general Amber force field (GAFF).^64^ Charges for the ligands were obtained with the restrained electrostatic potential method.^65^ The ligands were optimized with the semiempirical AM1 method, followed by a single-point calculation at the Hartree–Fock/6-31G* level to obtain the electrostatic potentials, sampled with the Merz–Kollman scheme.^66^ These calculations were performed with the Gaussian 09 software.^67^ The potentials were then used by antechamber to calculate the charges. A few missing parameters were taken from a previous study.^8^

For each complex, 1000 steps of minimization were used, followed by a 20 ps constant-volume equilibration and 20 ps constant-pressure equilibration, all performed with heavy nonwater atoms restrained toward the starting structure with a force constant of 4184 kJ/mol/Å^2^. Finally, the system was equilibrated for 1 ns, followed by 100 ns of production simulation, both performed with constant pressure and without any restraints. For each protein–ligand complex, 10 independent simulations were run.^68^ Consequently, the total simulation time for each complex was 1 μs. All bonds involving hydrogen atoms were constrained to the equilibrium value using the SHAKE algorithm,^69^ allowing for a time step of 2 fs. The temperature was kept constant at 301 K using Langevin dynamics,^70^ with a collision frequency of 2 ps^–1^. The pressure was kept constant at 1 atm using a weak-coupling isotropic algorithm^71^ with a relaxation time of 1 ps. Long-range electrostatics were handled by particle-mesh Ewald (PME) summation^72^ with a fourth-order B spline interpolation and a tolerance of 10^–5^. The cutoff radius for Lennard–Jones interactions between atoms of neighboring boxes was set to 8 Å. The snapshots were analyzed with the *cpptraj* module.^36^ We also performed simulations of the three ligands free in solution, using the same setup.

### Conformational Entropy Estimates from MD Simulations

We calculated NH order parameters from the MD trajectories using isotropic reorientational eigenmode dynamic analysis.^73^ The covariance matrix of the NH bond vectors was obtained from the trajectories by the *cpptraj* module^36^ in the Amber software.^58^

Conformational entropies were calculated from the ensemble of configurations of the protein and ligands by analyzing the dihedral angle fluctuations.^4,60,74,75^ The Cartesian coordinates from the trajectories were transformed to internal coordinates, and the entropies were then calculated from probability distributions over all possible states of these coordinates using a bin size of 5° (i.e., 72 bins per dihedral). Entropies were normalized to that of a free rotor.^4^ All entropies are reported as −*T*Δ*S* at 301 K. Protein-backbone entropies were calculated from two-dimensional distributions of the ϕ and ψ dihedral angles. In our previous study, we showed that the effect of correlated motions is minimal for entropy estimates of galectin-3C, less than 1 kJ/mol.^8^

A total of 100 000 snapshots with a 10 ps sampling frequency were used for entropy and order parameter estimates, employing separate simulations for the complexes, for free galectin-3C, and for the solvated ligands. Entropies and order parameters were calculated as averages over 200 simulations of 5 ns (with 500 snapshots in each, i.e., each of the 10 simulations was divided into 20 parts of equal length). The 5 ns time window is similar to the rotational correlation time of the protein (∼7 ns). This procedure yields more stable entropy estimates by restricting the dependence on rare events.^60^ The reported uncertainties are standard errors over these 200 simulations.

### Solvation Thermodynamics

We identified water sites and analyzed the thermodynamics of the solvent around the galectin-3C complexes, using GIST,^76,77^ implemented in the *cpptraj* module of the Amber software. The method requires snapshots from MD simulations in which the solute is kept restrained. Therefore, we performed 10 independent 100 ns long MD simulations, in which the solute was kept restrained toward the starting crystal structure (the unrestrained MD simulations did not show any extensive movements of the ligands or the protein, except for individual side chains).

For each protein–ligand system, the water–water interaction energy, *E*_ww_, and solute–water interaction energy, *E*_sw_, as well as translational, *S*_trans_, and rotational, *S*_rot_, entropy contributions were calculated for a rectangular grid of dimensions 24 × 14 × 14.5 Å, centered on the ligand and extended at least 3 Å on each side of the ligand; for the free ligands, the dimensions were 16.5 × 18 × 21 Å. The grid was divided into cubic boxes (0.5 × 0.5 × 0.5 Å), for which the thermodynamic properties were calculated. The sum of these properties over the entire region reveals the changes in the hydration thermodynamics of the region for each cluster, relative to bulk water.

Solvation free energies for the free ligands in water solution were calculated with the conductor-like solvent model (COSMO)^78,79^ real-solvent (COSMO-RS) approach^80,81^ using the COSMOTHERM software.^82^ This is one of the most accurate methods to estimate solvation free energies of small molecules.^83,84^ The calculations were based on two single-point density-functional theory calculations with the BP86 method^85,86^ and the TZVP basis set,^87^ one performed in a vacuum and the other in the COSMO solvent with an infinite dielectric constant, as required by method. The structures were taken from unrestrained MD simulations of the free ligands, 110 snapshots from each ligand.

## Results and Discussion

### Ligand Design

**Figure 1:**
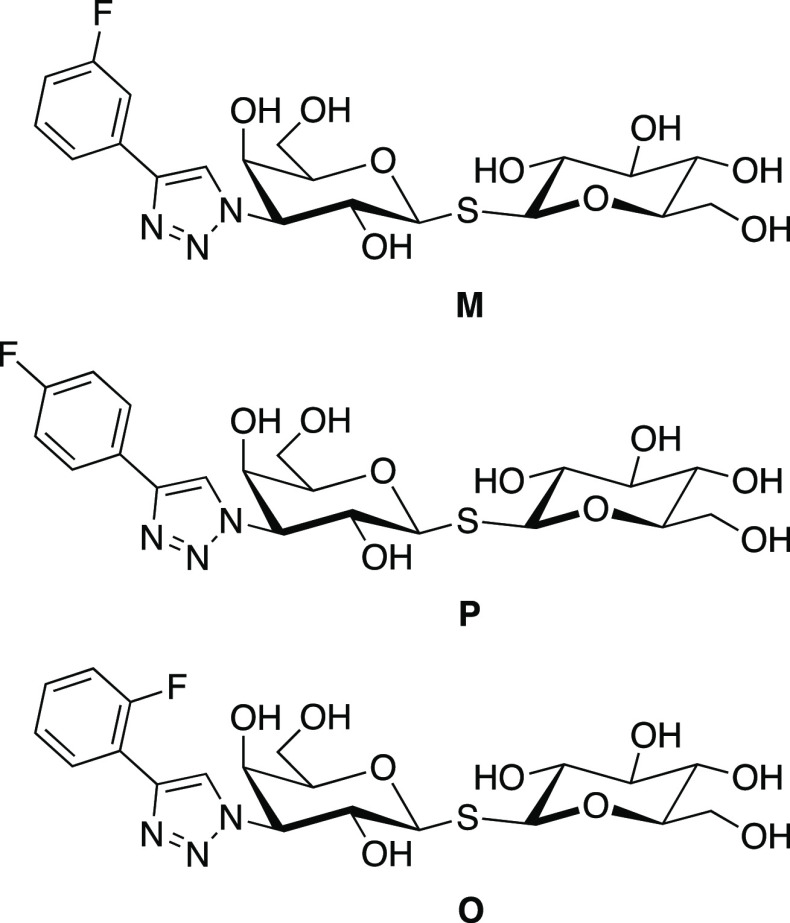
Chemical structures of the three fluorinated phenyltriazolyl-galactosylthioglucoside galectin-3C ligands M, P, and O.

We synthesized *o*-, *m*-, and *p*-fluoro-phenyltriazolyl-galactosylthioglucosides (O, M, and P; see Figure 1) following protocols published recently.^23^ The present series of ligands includes a glucose unit, instead of the *S*-toluene group used in previous studies,^23^ in order to make the ligands water-soluble without the addition of DMSO.

### Overall Binding Thermodynamics

**Figure 2:**
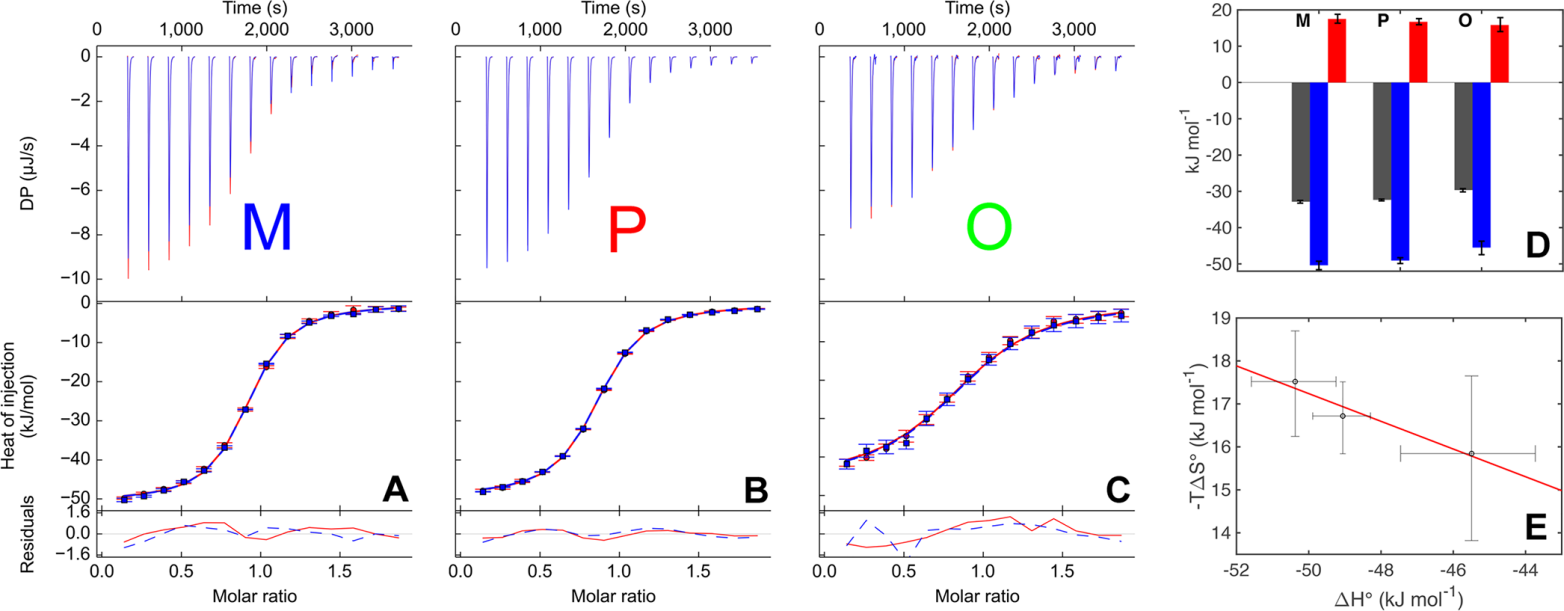
ITC experiments of ligand binding to galectin-3C. (A–C) Raw thermograms showing the differential power (upper panels) and isotherms with residuals of the fits (lower panel). Duplicate data sets are shown in red and blue. The ligands are ordered left to right according to their affinity. The concentrations of ligand and protein are identical in the different experiments, and the isotherms are therefore directly comparable. (D) Thermodynamic profiles of the three complexes with Δ*G*° (dark gray), Δ*H*° (blue), and −*T*Δ*S*° (red). (E) Graph of −*T*Δ*S*° versus Δ*H*° suggesting enthalpy–entropy compensation. The error bars indicate a very modest asymmetry, as determined by the ITCsy/SEDPHAT software. The adjusted *R*^2^ correlation coefficient is 0.89, and the *p* value is 0.15, showing that the statistical confidence for a linear relation between Δ*H*° and −*T*Δ*S*° is quite low.

We characterized the overall macroscopic thermodynamics for the binding of the three ligands to galectin-3C using ITC. We performed two replicate titrations with each of the ligands, followed by a combined fit of the duplicate data (Figure 2A–C). Table 1 lists the resulting thermodynamic parameters, which are also summarized in Figure 2D. The resulting dissociation constants fall in the low micromolar range, *K*_d_(M) = 2.0 ± 0.3 μM, *K*_d_(P) = 2.5 ± 0.2 μM, and *K*_d_(O) = 7.2 ± 1.3 μM. Similar to our previous observations for other ligand series,^5,8^ the relative affinities correlate well with results from competitive fluorescence polarization experiments. The Δ*H°* values for M– and P–galectin-3C are −50.4 ± 1.2 and −49.1 ± 0.8 kJ/mol, hence equal within error, while O–galectin-3C has a smaller enthalpic contribution of −45.5 ± 1.9. The entropies (−*T*Δ*S*°) are 17.5 ± 1.2, 16.7 ± 0.8, and 15.8 ± 1.9 kJ/mol for the complexes with M, P, and O, respectively. The data follow a trend suggesting enthalpy–entropy compensation (Figure 2E), as often seen in ligand–protein interactions, but the association between Δ*H*° and −*T*Δ*S*° is not statistically significant (*p* = 0.15). Taken together, the ITC experiments reveal that the complexes have similar thermodynamic signatures overall, which might be expected from the similar structure of the ligands. The samples resulting from the ITC titrations were characterized by ^1^H–^15^N HSQC spectroscopy and compared to the separately prepared NMR samples, to make sure that the protein chemical shifts were identical in the two samples, thereby verifying that the sample conditions and protein characteristics were virtually identical in the two types of experiments. Based on the determined *K*_d_ values and reactant concentrations, the saturation levels of the complexes after the last ITC injection were 97.6, 97.0, and 92.4% for M, P, and O, respectively.

**Table 1:** Overall Binding Thermodynamics from ITC

complex	*K*_d_ ITC (10^–6^ M)^a^	Δ*G*°_tot_ (kJ/mol)^a^	Δ*H*°_tot_ (kJ/mol)^a^	–*T*Δ*S°*_tot_ (kJ/mol)^a^
M	2.00 ± 0.29	–32.9 ± 0.4	–50.4 ± 1.2	17.5 ± 1.2
P	2.46 ± 0.24	–32.3 ± 0.3	–49.1 ± 0.8	16.7 ± 0.8
O	7.21 ± 1.28	–29.6 ± 0.4	–45.5 ± 1.9	15.8 ± 1.9

a Reported errors are averages of the asymmetric upper and lower error bounds (1 SD) given by ITCsy/SEDPHAT, which are shown Figure 2E.

### Crystal Structures Reveal Subtle Differences in Binding Modes

**Figure 3:**
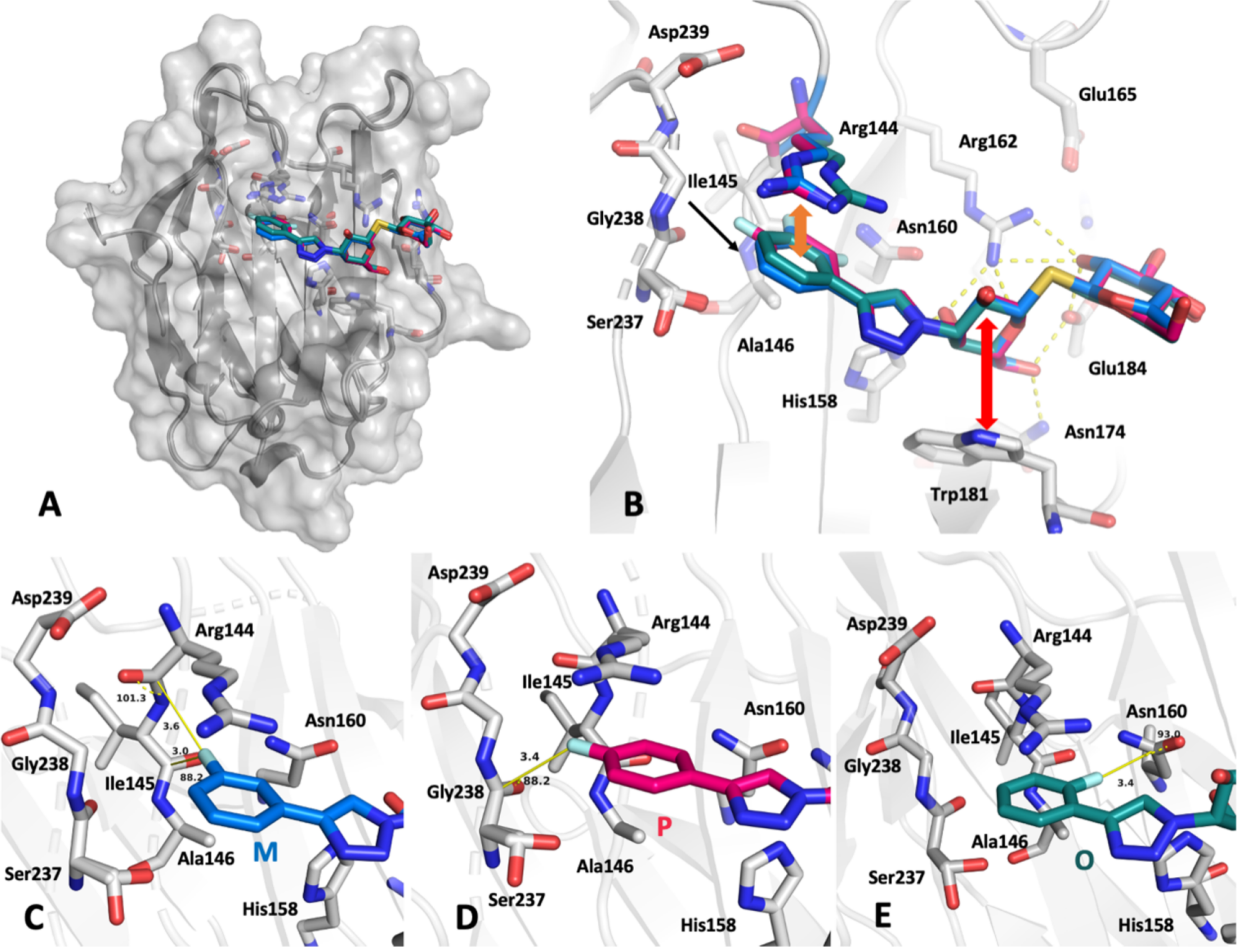
X-ray crystal structures of the ligand–galectin-3C complexes. (A) Surface view of the protein with all three ligands shown as sticks (O, M, and P colored green, blue, and red, respectively). (B) Overview of the binding site with the ligands superimposed. (C–E) Close-up view of the fluorophenyl moiety of the M (C), P (D), and O (E) ligands. The protein backbone is shown in ribbon representation (gray), whereas key ligand-coordinating backbone segments and side chains are shown as sticks. In panel B, key interactions are indicated: hydrogen bonds involving the ligand are shown as dashed yellow lines, the π–π interaction involving Arg144 and the phenyl group of the ligand is shown as an orange double arrow, and the CH−π stacking interaction involving Trp181 and the galactose moiety is shown as a red double arrow. In panels C–E, distances and angles of the fluorine–amide interactions are indicated and depicted in yellow as solid lines and dashed semicircles, respectively. Note that the side chain of Ser237 is modeled in two conformations.

Very high-resolution crystal structures were obtained for galectin-3C in complex with the three ligands (resolution = 0.94–1.01 Å, cf. Table S1). The ligands are very well-defined by the electron density (Figure S1). The overall binding mode of the ligands (Figure 3A) is identical to that of previously reported analogous phenyltriazolyl-thiodigalactosides.^23^ The galactose moiety forms a CH−π stacking interaction with Trp181 and the glucose HO2 forms hydrogen bond interactions with Arg162, Glu184, and Arg186 (Figure 3B).^13,88^ Arg144 forms a cation−π (or π–π) interaction with the phenyl group.^89^ The orientation of the phenyl ring differs subtly with the position of the fluorine (Figure 3B). The fluorophenyltriazole moiety has polar interactions with the backbone carbonyl moieties of Arg144, Ile145, Asn160, Ser237, and Gly238. Fluorine atoms tend to orient toward nearby backbone amides, side-chain amides, and carbonyl carbons in an orthogonal manner.^90−92^ The geometry of these interactions depends on the distance to the peptidic NH and CO groups, such that the angle tends toward 90° at shorter distances. Figure 3C–E shows how the fluorines are oriented toward the neighboring carbonyl moieties. The fluorine in M forms an interaction with the carbonyl moieties of Arg144 and Ile145. The fluorine in P interacts with the carbonyl moiety of Ser237. For the fluorine in O, there is no peptide backbone nearby. Instead, it orients toward the carbonyl group of the Asn160 side chain. However, recent quantum-mechanical calculations have indicated that these fluorine–amide interactions have little influence on the binding affinities (the fluorine atom does not form stronger amide interactions than a phenyl hydrogen atom).^93^ The electron density of the fluorophenyl group in O is slightly less well-defined (Figure S1), which likely is due to increased mobility. Possibly, the higher mobility of O could be related to the fluorine being coordinated by a side chain rather than a backbone carbonyl as in the case of M and P. The positions and occupancies of water molecules around the binding site are quite similar for all three ligands. Complete data collection and refinement statistics are listed in Table S1 of the Supporting Information.

### Ensemble Refinement of Crystal Structures Highlights Differences in Flexibility

**Figure 4:**
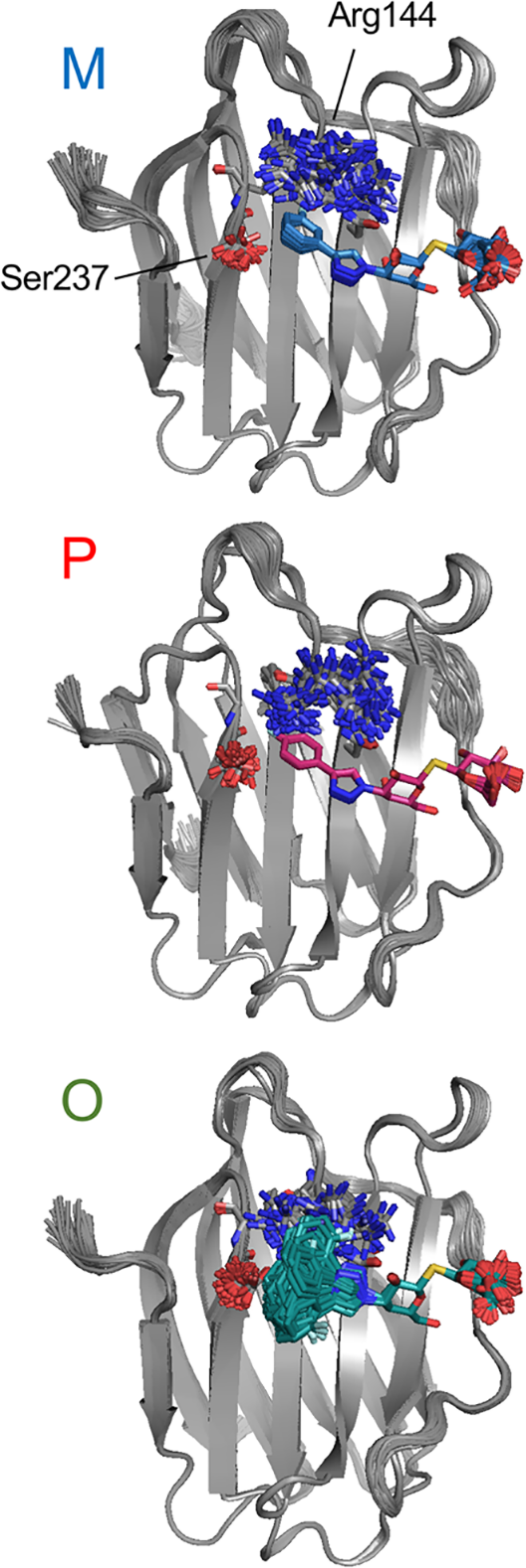
Ensemble-refined X-ray crystal structures. Overlay of the best 100 structures (in terms of the *R* factor) generated by ensemble refinement for M (blue), P (red), and O-bound (green) galectin-3C. Two residues that show large movement in all three complexes are shown in stick representation: Ser237 with its side-chain oxygen colored red and Arg144 with its side-chain nitrogens colored blue.

We carried out ensemble refinement of the three complexes in order to compare their flexibility in the crystal. The results are complementary to the order parameters measured by NMR relaxation and to the MD simulations (described below). The resulting ensembles show what conformations are compatible with the crystallographic raw data and may point out groups that show larger movements than indicated by the crystallographic *B* factors. These coordinate distributions can be translated into conformational entropies, although not quantitatively.^94^Figure 4 shows that the M and P ligands exhibit only one conformation in the crystal structure, with M having slightly higher fluctuations. In contrast, O exhibits a relatively high degree of mobility of the fluorophenyl moiety. The glucose ring is also flexible in all three complexes, most so for O and least so for P. This indicates a higher conformational entropy of O in the complex. However, the protein atoms show the opposite behavior, with a slightly higher root-mean-square fluctuation (RMSF) for the M and P complexes (RMSF = 0.06 ± 0.01 Å for both complexes), whereas the O complex has a lower RMSF of 0.053 ± 0.01 Å. Individual protein residues show varying degrees of flexibility. Among those interacting with the ligands, Arg144 is the most flexible. This residue interacts through π–π stacking with the fluorophenyl ring in the ligands, but its flexibility is not significantly different between the three complexes. The side chain of Ser237, which is modeled in two alternative conformations in the crystal structures of all three complexes, also shows multiple conformations in the ensemble refinement simulations of all three complexes. The carbonyl groups that interact with the fluorine atoms of the ligands, viz., the Asn160 side chain for O, the Ile145 backbone for M, and the Ser237 backbone for P, do not show any significant flexibility in any of the three complexes.

### Chemical Shift Mapping of Ligand Binding

Differences in chemical shifts between protein–ligand complexes provide a sensitive indication of differences in structure and dynamics. By mapping chemical shift changes in the protein upon ligand binding, we verify that the ligands bind to the protein in solution in the same pose as that observed in the crystal structures. In addition, chemical shift differences between complexes serve to validate results of the ensemble-refined X-ray structures and can further identify subtle differences between the complexes in their structure and dynamics.

**Figure 5:**
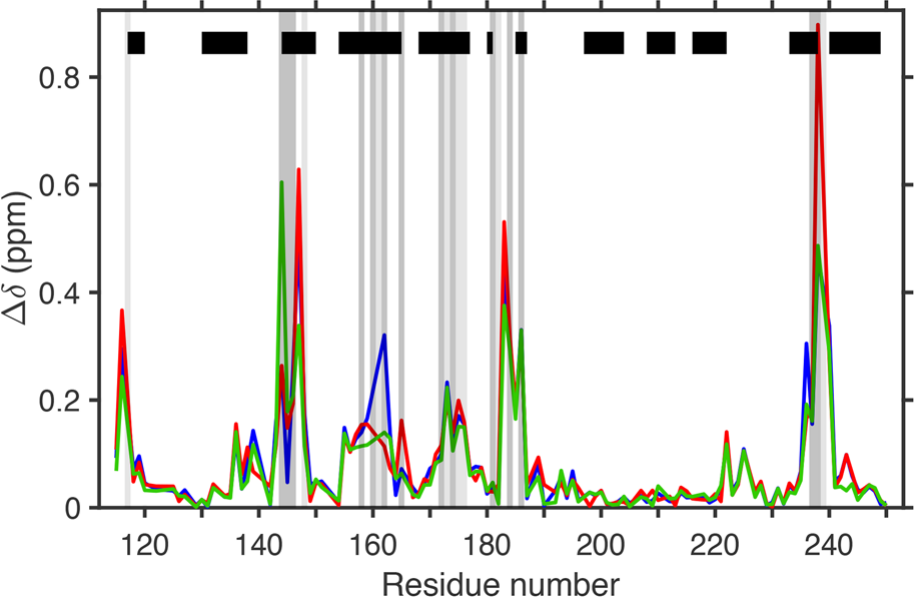
Chemical shift perturbations upon ligand binding. Backbone amide chemical shift differences relative to apo galectin-3C are shown for M–, P–, and O–galectin-3C in blue, red, and green, respectively. The black horizontal bars represent the β-sheet secondary structure of galectin-3C, and the gray vertical bars indicate residues with any atom closer than 4 Å (dark gray) or 6 Å (light gray) from any ligand atom.

We assigned the chemical shifts of O–, M–, and P–galectin-3C based on HNCACB and CACB(CO)NH experiments and assignments reported previously.^4,5,8^ Superimposed spectra of the three complexes are shown in the SI (Figures S2 and S3). In keeping with previous results,^5,8^ ligand binding leads to chemical shift differences for the backbone amides throughout the protein. The chemical shifts of 115 backbone amides were used to monitor ligand binding (Figure 5; intercomplex comparisons are shown in Figure S4A). Significant chemical shift differences in the ^1^H–^15^N HSQC spectra between the three complexes are observed primarily in regions close to the ligand (identified by gray vertical bars in Figures 5 and S4B), including residues 144–147, 158–165, 173–175, 184, and 235–239 but also for residues further away, viz., residues 116, 139, and 243.

In several cases, the observed chemical shift differences among the three complexes can be rationalized in terms of structural differences observed in the crystal structures. Starting with residues to the left in the binding site as presented in Figure 3B, we notice that the backbone NH of Gly238 in the P complex shows the largest chemical shift perturbation of all residues. This effect is explained by the interaction between the carbonyl of Ser237 and the fluorine atom with an interatomic distance of 3.4 Å (Figure 3D). The NH shift of Gly238 is perturbed in the M and O complexes as well but not to the same extent. The backbone NH shift of Arg144 in the O complex differs considerably from the shift in the M and P complexes. This difference might reflect the average orientation of the Arg144 side chain, which is different in the O complex as compared to the M and P complexes (Figure 3B). The backbone NH chemical shifts of Ile145, Ala146, and Leu147 all show significant variation among the three complexes that likely can be explained in part by differences in the interactions between the fluorine and these amide groups. In the case of Ile145, the distance between the fluorine and the carbonyl group of the preceding residue varies from 3.6 Å in the M complex to 4.4 and 5.8 Å in the P and O complexes, respectively. As for Ala146, the corresponding distances are 3.0 Å (M), 4.1 Å (P), and 4.7 Å (O). Leu147 is further removed from the fluorine atoms with a distance greater than 5 Å in each complex, suggesting that variable fluorine interactions alone do not explain the observed variation in chemical shift. Rather, an additional contribution is expected from changes in the orientation of the fluorophenyl moiety (Figure 3B), which causes significant ring current shifts of the surrounding residues.

A number of other residues show NH chemical shift differences between complexes that cannot be explained by the static structures, which are virtually identical in these regions. Most likely, these shift changes report on differential dynamic averaging of the chemical shifts. Prominent examples include Arg162 and Glu165, the side chains of which pack together and interact with the glucose moiety, as seen in Figure 3B. These results reflect the differences in flexibility of the glucose ring observed in the ensemble-refined X-ray structures.

Chemical shift differences are also observed for methyl groups (Figure S4B), but there are few methyl-bearing side chains close to binding site. Notably, Ala146 Cβ shows the largest perturbation by far. The methyl group of Ala146 is located directly beneath the fluorophenyl ring (Figure 3C–E). Thus, the chemical shift of this methyl group is exquisitely sensitive to the ring orientation (cf., Figure 4) and the fluorine position. Val116 has the second-most perturbed methyl group, which is directed toward the fluorophenyl ring, located some 7 Å away. There is no difference in structure in this region among the complexes. However, we note that the backbone amide of Gly238 is positioned between Cγ1 of Val116 and the fluorophenyl group. Given that the NH of Gly238 has the most perturbed chemical shift, it might also be expected to find large perturbations for the Val116 side chain.

### Conformational Fluctuations Measured by NMR

We performed a series of protein NMR relaxation experiments to characterize the conformational dynamics on the pico- to nanosecond time scale. In the following analysis, we include only residues for which data are available for all three complexes, comprising 116 out of the 138 backbone amides and 73 out of the 85 methyl groups. The missing residues had cross-peaks that were overlapped or too broadened to allow for quantitative analysis.

We interpreted the relaxation data using the model-free formalism.^47,48^ The best-fit rotational diffusion tensor determined from the backbone relaxation data is anisotropic for all three complexes, with ζ values of 1.11, 1.08, and 1.08 for the M, P, and O complexes, respectively. The P complex has an axially symmetric diffusion tensor, whereas the M and O complexes have rhombic tensors with η values of 0.54 and 0.75, respectively. The correlation times (τ_c_) are 7.12, 6.97, and 6.95 ns for M, P, and O. ^15^N relaxation data back-calculated from the optimized model-free parameters show good agreement with the experimental values for all three complexes (Figures S5–7).

In addition, we characterized the dynamics of the fluorinated phenyl rings of the bound ligands using ^19^F *R*_2_ relaxation experiments. The results reveal that the ^19^F *R*_2_ is significantly enhanced for the O ligand compared to the other two ligands, indicating that the O ligand undergoes conformational exchange on a fast time scale (Figure S8). In contrast, the M and P ligands both appear to populate a single conformation in the complex. Notably, these results are in perfect agreement with the ensemble-refined X-ray crystal structures (cf., Figure 4).

### Differences in Backbone Order Parameters

**Figure 6:**
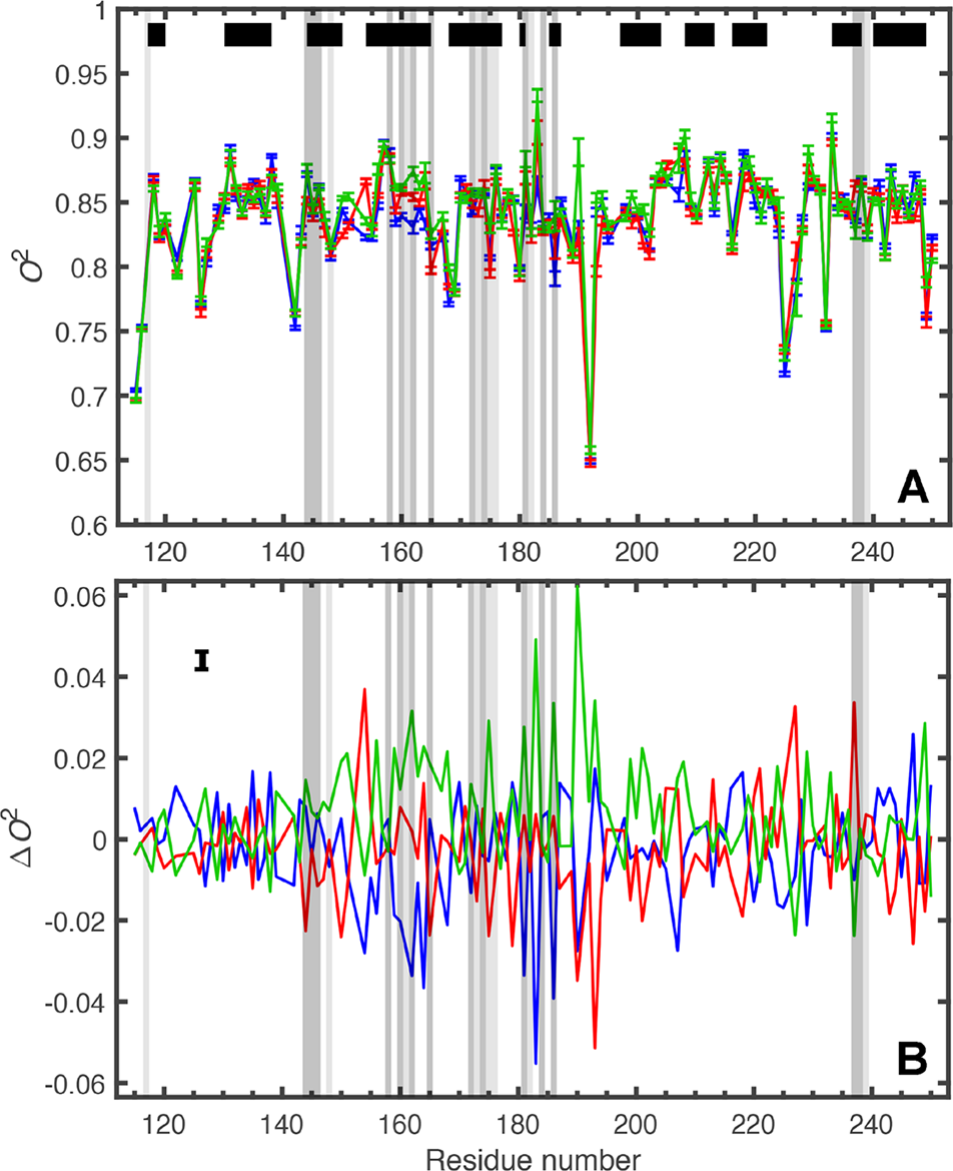
Comparison of the backbone order parameters for the three ligand–galectin-3C complexes. (A) Backbone order parameters for M– (blue), P– (red), and O–galectin-3C (green). (B) Backbone intercomplex Δ*O*^2^ values. Error bars are removed to improve clarity, but a representative error bar, corresponding to the mean error, is placed in the upper left corner. The same 116 residues are shown for each complex. The black and gray bars have the same meaning as in Figure 5.

The backbone order parameters are very similar among the three complexes (Figure 6A). Backbone *O*^2^ values are listed in Table S2, and their distributions are represented as histograms in Figure S9. The mean values and standard error of the means are <*O*^2^> = 0.837 ± 0.004, 0.837 ± 0.004, and 0.843 ± 0.004 for M–, P–, and O–galectin-3C, respectively. There is no significant difference between the mean values, as gauged by one-way ANOVA (*p* = 0.41). The subtle differences in *O*^2^ revealed by the intercomplex comparisons (Figure 6B; pairwise comparisons are shown in Figure S10) might be expected based on the small structural differences between the complexes. It is clear that M– and P–galectin-3C have more flexible residues close to the binding site than does O–galectin-3C, which is also reflected in the slightly higher <*O*^2^> for the O complex. These results are in qualitative agreement with RMSF values determined from the ensemble-refined X-ray structures (cf., Figure 4).

The high degree of similarity among the three complexes in *O*^2^ for residues outside of the binding region attests to the high reproducibility of the relaxation rate measurements and indicates that statistically significant differences in *O*^2^ can be meaningfully interpreted in terms of conformational entropy differences. The main differences in the backbone *O*^2^ are seen for residues at the binding site (Figures 6B and S10). Among these, subtle differences in *O*^2^ are observed in the segment 159–165, which includes several residues that are either in direct contact with the ligand (Asn160 and Arg162) or participating in the hydrogen-bonding network around the ligand (Glu165). Most likely, variation in side-chain interactions with the ligand among the different complexes affects the backbone fluctuations. Further, residues Thr175, Asn179, Trp181, Arg186, and Ser237 also show differences in *O*^2^. Trp181 and Arg186 contact the ligand with their side chains, while Thr175 forms a hydrogen bond with Gly182. The carbonyl of Ser237 is involved in a fluorine–amide interaction in the P complex, which shows the highest *O*^2^, whereas *O*^2^ is lowest in the O complex for this residue. The largest differences are observed for Arg183, Phe190, and Glu193, none of which are located within 6 Å from the ligand. All three backbone amides lack hydrogen bonding partners and appear in nonregular secondary structure elements. Despite being located in a more remote β-turn, residues 150–154 also show minor differences that again might arise from variations in ligand interactions relayed from the binding site via a network of side chains, see further below.

**Figure 7:**
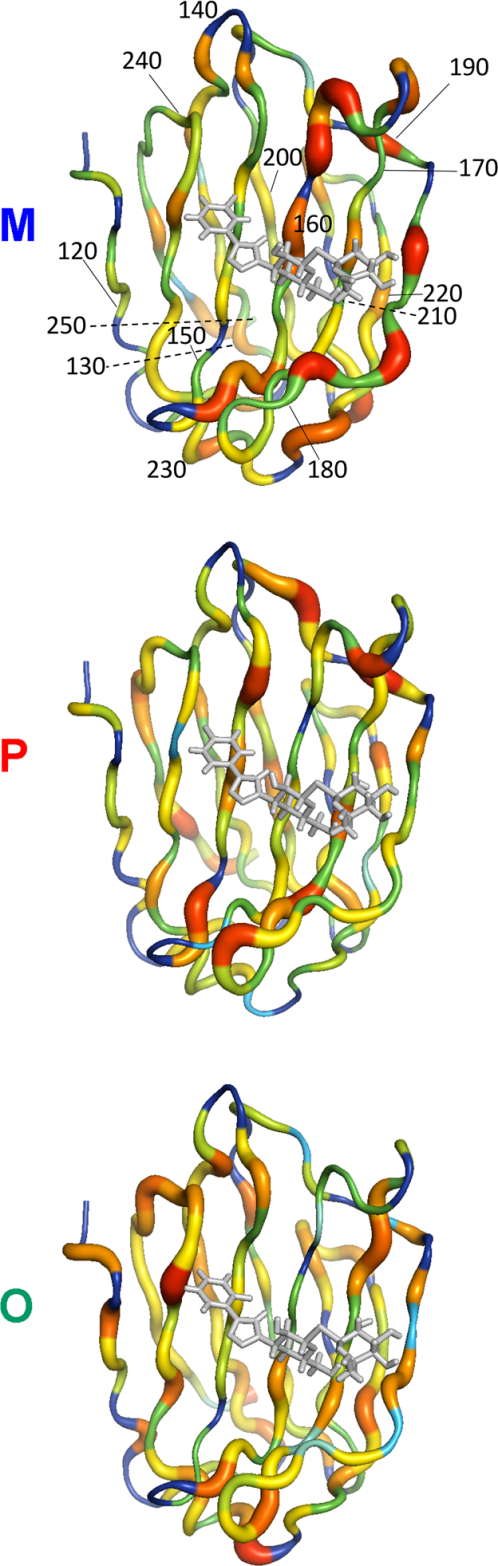
Intercomplex comparison of backbone order parameters. Δ*O*^2^ is color- and width-coded onto a tube representation of the protein backbone. Thin cyan tubes have Δ*O*^2^ ≥ 0.025 (indicating less flexibility), followed by green, yellow, orange, and the wide red tubes, which have Δ*O*^2^ ≤ −0.025 (indicating more flexibility). Dark blue indicates residues for which data are missing. The ligand is shown in gray. For reference, every 10th residue is marked in the M complex.

The intercomplex differences in backbone order parameters are mapped onto the structures in Figure 7, where red, wide tubes represent residues that are more flexible (Δ*O*^2^ ≤ −0.025) in the given complex compared to the other two complexes, while cyan, thin tubes indicate residues that are less flexible (Δ*O*^2^ ≥ 0.025). Residues that have greater flexibility in M–galectin-3C than in the other two complexes are found very close to the binding site (<3 Å away), see Figure 7. In P–galectin-3C, residues belonging to the corresponding category are located somewhat further away (<5 Å) from the binding site (Figure 7). In addition, the M and P complexes both have additional residues with Δ*O*^2^ ≤ −0.02 (wide orange or red tubes in Figure 7) located within 11 Å of the binding site. In O–galectin-3C, the only residues with Δ*O*^2^ ≤ −0.02 are Ser237 in the binding site and Lys227, which is located 18 Å from the ligand (Figure 7).

### Differences in Side-Chain Order Parameters

**Figure 8:**
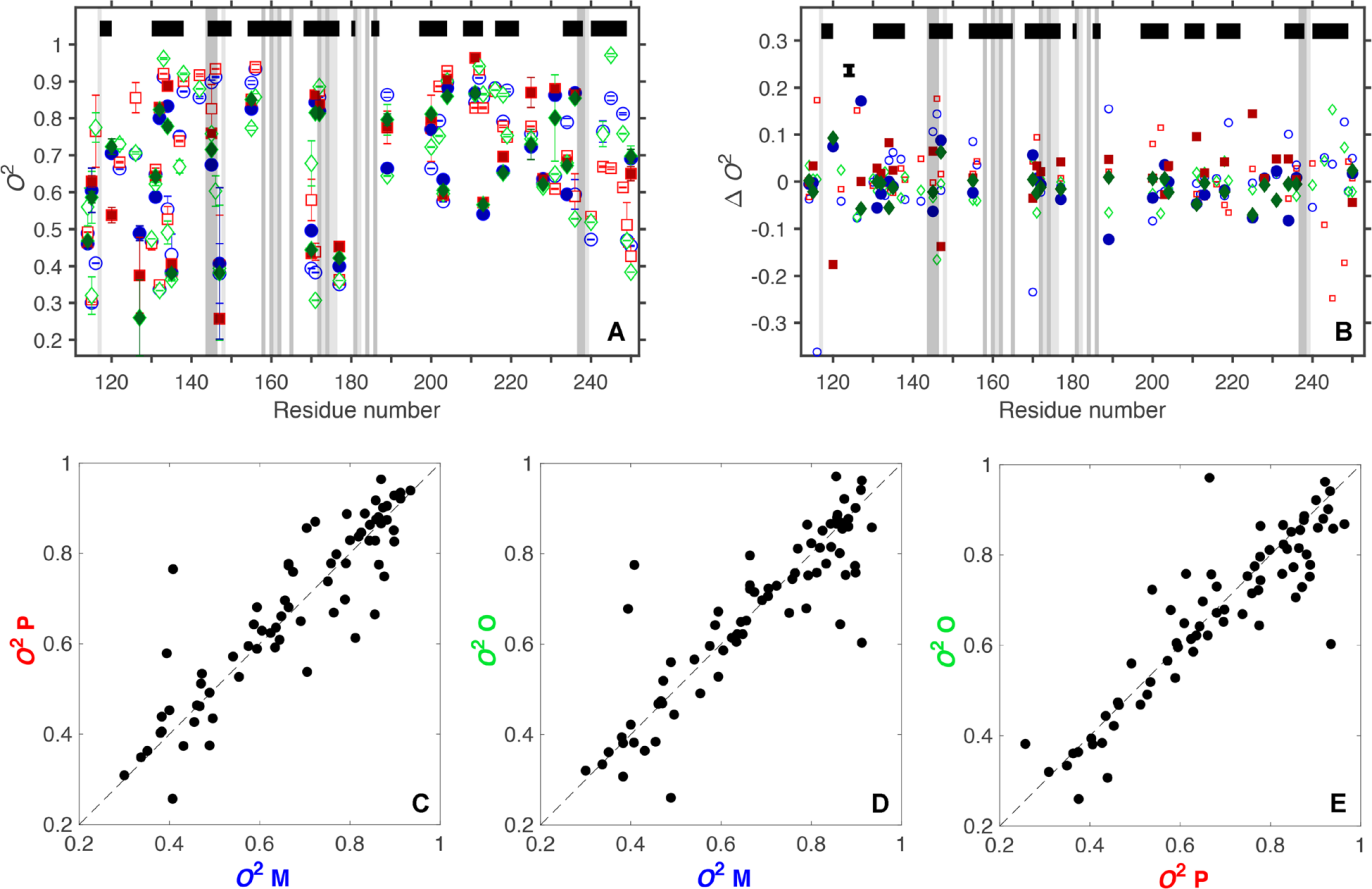
Comparison of methyl-axis order parameters for the various ligand–galectin-3C complexes. (A) O– (green diamonds), M– (blue circles), and P–galectin-3C (red squares) order parameters, reported for 73 methyl groups. Open markers represent residues with a single methyl group (Ala, Thr, and Met) and methyl groups Cγ1 (Val), Cδ1 (Leu), or Cδ1 (Ile), whereas filled markers refer to Cγ2 (Val), Cδ2 (Leu), or Cγ2 (Ile). Error bars indicate 1 SD of (B) methyl Δ*O*^2^ values representing intercomplex comparisons. For clarity, individual error bars are not shown, but an error bar representing the mean error is placed in the upper left-hand corner. (C–E) Correlation plots of the data presented in panel (A); the dotted lines have a slope of 1.

The side chains are naturally more flexible than the backbone, as reflected by their generally lower order parameters. The order parameters are listed in Table S3, and Figure S9 shows histograms of the *O*^2^ distributions for each complex. The mean values and standard error of the means are <*O*^2^> = 0.675 ± 0.022, 0.684 ± 0.022, and 0.670 ± 0.021 (*N* = 73) for M–, P–, and O–galectin-3C, respectively. As in the case of the backbone order parameters, one-way ANOVA does not reject the null hypothesis of equal means. Figure 8 presents an overview of the residue-specific values of *O*^2^ as well as the intercomplex differences, Δ*O*^2^, while Figure S11 shows the pairwise comparisons.

We investigated the detailed differences observed for individual residues between the complexes. There are only 5 methyl-containing residues among the 28 residues within 6 Å of the ligand, where the greatest variations among complexes in backbone order parameters are seen. Side-chain ^2^H relaxation data are available for four of these residues, namely Ile145, Ala146, Leu147, and Val172. Among these, Ala146 shows the greatest variation with *O*^2^ values of 0.92, 0.93, and 0.60 for the M, P, and O complexes, respectively. Interestingly, Ala146 interacts directly with the fluorinated phenyl ring of the ligand. The higher flexibility of Ala146 in the O complex is in agreement with the ensemble-refined crystal structure and the ^19^F relaxation data, which both indicate significantly greater fluctuations of the fluorophenyl ring in the O complex than in the other two complexes (cf., Figure 4). Clearly, the Ala146 methyl group senses the fluctuations of the ring. Ile145 Cγ1 also shows a lower *O*^2^ of 0.76 in the O complex than in the other two complexes (0.90 and 0.83 for M and P). Ile145 Cδ2 shows *O*^2^ values of 0.67, 0.76, and 0.72 for the M, P, and O complexes. Leu147 does not show any significant differences in *O*^2^, and Val172 shows only very minor differences.

Outside of the immediate binding site, Val116 Cγ1 also shows significant differences with *O*^2^ of 0.41, 0.77, and 0.78 for the M, P, and O complexes (data are not available for Val116 Cγ2 due to spectral overlap). Ala146 and Val116 are located on either side of Ser237, the backbone of which responds to differences in the fluorine position (as described above). Further, Val155 Cγ1 and Ala156 show minor Δ*O*^2^, as was also observed for the backbone of residues 150–154. The side chain of Val225, which packs against Ala156, also exhibits a difference between complexes with Cγ2 having a higher *O*^2^ for the P complex. Leu120, Leu234, and Ala245 form a cluster of interacting side chains that all show minor Δ*O*^2^. Both Leu120 Cδ2 and Ala245 have the lowest *O*^2^ in the P complex, but these results cannot be explained by inspecting the crystal structures. Further away from the binding site, Val126 Cγ1 shows the opposite behavior compared with Leu120 and Ala 245. Finally, Val170 Cγ1, which packs against V172, has *O*^2^ values of 0.39, 0.58, and 0.68 for the M, P, and O complexes, respectively. Similar to previous results for a number of galectin-3C complexes,^5,8^ differences in order parameters do not generally correspond to obvious structural differences between the complexes. This finding reconfirms that subtle but statistically significant differences in dynamics can arise among structurally similar complexes.

### Conformational Entropy Differences Determined by NMR

The nonlinear dependence of entropy on the backbone order parameter, together with residue-specific variations in Δ*O*^2^, lead to significant differences in entropy between complexes, despite <*O*^2^> not showing significant variation among M–, P–, and O–galectin-3C. We estimated the differences between complexes in their conformational entropy contributions to ligand binding, using eqs 1–3 (Table 2). M– and P–galectin-3C have almost identical conformational backbone entropy, yielding intercomplex differences of −*T*ΔΔ*S*_bb,M_ = −5.7 ± 0.6 kJ/mol and −*T*ΔΔ*S*_bb,P_ = −6.0 ± 0.8 kJ/mol, whereas O–galectin-3C has a lower backbone entropy yielding −*T*ΔΔ*S*_bb,O_ = 11.7 ± 0.8 kJ/mol. Hence, the backbone conformational entropy favors the binding of M and P, whereas the binding of O is disfavored in the intercomplex comparison. The side-chain entropy differences are less pronounced and have greater uncertainty: −*T*ΔΔ*S*_sc,M_ = −0.4 ± 2 kJ/mol, −*T*ΔΔ*S*_sc,P_ = 4 ± 2 kJ/mol, and −*T*ΔΔ*S*_sc,O_ = −4 ± 2 kJ/mol. The empirically calibrated “entropy meter”, proposed by Wand and co-workers,^95^ is based on the difference between complexes in their average order parameter of the methyl axes and consequently does not capture the minor effects observed here, because <*O*^2^> is not significantly different between the M, P, and O complexes.

**Table 2:** Intercomplex Differences in Protein Conformational Entropy Estimated from NMR Order Parameters

	–*TΔΔS*_conf_ (kJ mol^–1^)		
complex	bb^a^	sc^b^	bb + sc
M	–5.7 ± 0.6	–0.5 ± 2	–6 ± 2
P	–6.0 ± 0.8	4 ± 2	–2 ± 2
O	11.7 ± 0.8	–4 ± 2	8 ± 2

a Backbone: based on 116 backbone NH groups, errors are given as ±1 SD.

b Side chains: based on 73 methyl groups, errors are given as ±1 SD.

Summing up the conformational entropy of the backbone and the side chains results in intercomplex differences: −*T*ΔΔ*S*_bb+sc,M_ = −6 ± 2 kJ/mol, −*T*ΔΔ*S*_bb+sc,P_ = −2 ± 2 kJ/mol, and −*T*ΔΔ*S*_bb+sc,O_ = 8 ± 2 kJ/mol. The differences between M and P are not significant, while O differs significantly from the other two. This suggests that the conformational entropy of the backbone and methyl-bearing side chains determined by NMR favors binding of M and, to a minor extent, also P, whereas it disfavors binding of O.

### Conformational Fluctuations and Entropy Differences Determined by MD

MD simulations can provide information on the total conformational entropy of the protein and ligand. Therefore, we performed MD simulations to complement the entropies estimated from the NMR experiments, which are limited to sampling the backbone and methyl-bearing side chains.

The coordinate RMSFs from the MD simulations provide a first overview of differences between the complexes. The MD simulations confirm the results from the ensemble-refined crystal structures and the NMR data, showing that the RMSF is largest for the O ligand in the complex (1.16 ± 0.08 Å) and smallest for P (0.90 ± 0.09 Å). However, the RMSF for the protein barely differs between complexes, where P–galectin-3C has 0.84 ± 0.01 Å compared to 0.85–0.86 ± 0.01 Å for the M and O complexes.

To further validate the simulations against experimental data, we calculated order parameters for the backbone and compared with those measured by NMR. The simulated order parameters reproduce the experimental ones with a pairwise RMSD between 0.043 and 0.056, which is in line with previous results.^4,8,96^ The simulated order parameters are modestly higher than the experimental parameters on average, with <*O*^2^>_MD_ – <*O*^2^>_NMR_ = 0.006, 0.013, and 0.007 for the M, P, and O complexes, respectively. However, there are clear discrepancies in the details (Figure S12). Clearly, it is a great challenge for MD simulations to reproduce the variations in dynamics based on the minor differences in structure among the three complexes.

We estimated the conformational entropy from dihedral angle distributions in the MD trajectories. The MD simulations do not yield any clearly significant difference between the three complexes in the conformational entropy of the protein (Table S4) but suggest that it is slightly higher in the M complex, in line with the NMR results. However, the MD simulations do indicate a significant difference in the conformational entropy of the ligands within the complexes, where O deviates from M and P (Table S4). The ligand contributions to the total entropy change are 16.1 ± 0.4 kJ/mol for O, 19.6 ± 0.4 kJ/mol for M, and 20.4 ± 0.3 kJ/mol for P. The less unfavorable entropy contribution from the O ligand primarily reflects a higher degree of flexibility in the bound state, which agrees with the ensemble-refined crystal structures (Figure 4), the ^19^F relaxation data (Figure S8), and the lower order parameters of methyl-bearing side chains located in direct proximity to the ligand, mentioned above. By contrast, the conformational entropies of the free ligands agree within 1 kJ/mol (Table S5). Similar, but less reliable, results are obtained by molecular mechanics with generalized Born and surface area solvation (MM/GBSA) and interaction entropy calculations (Table S6); see the Supporting Information for details.

### Radial Distribution of Conformational Entropy around the Binding Site

**Figure 9:**
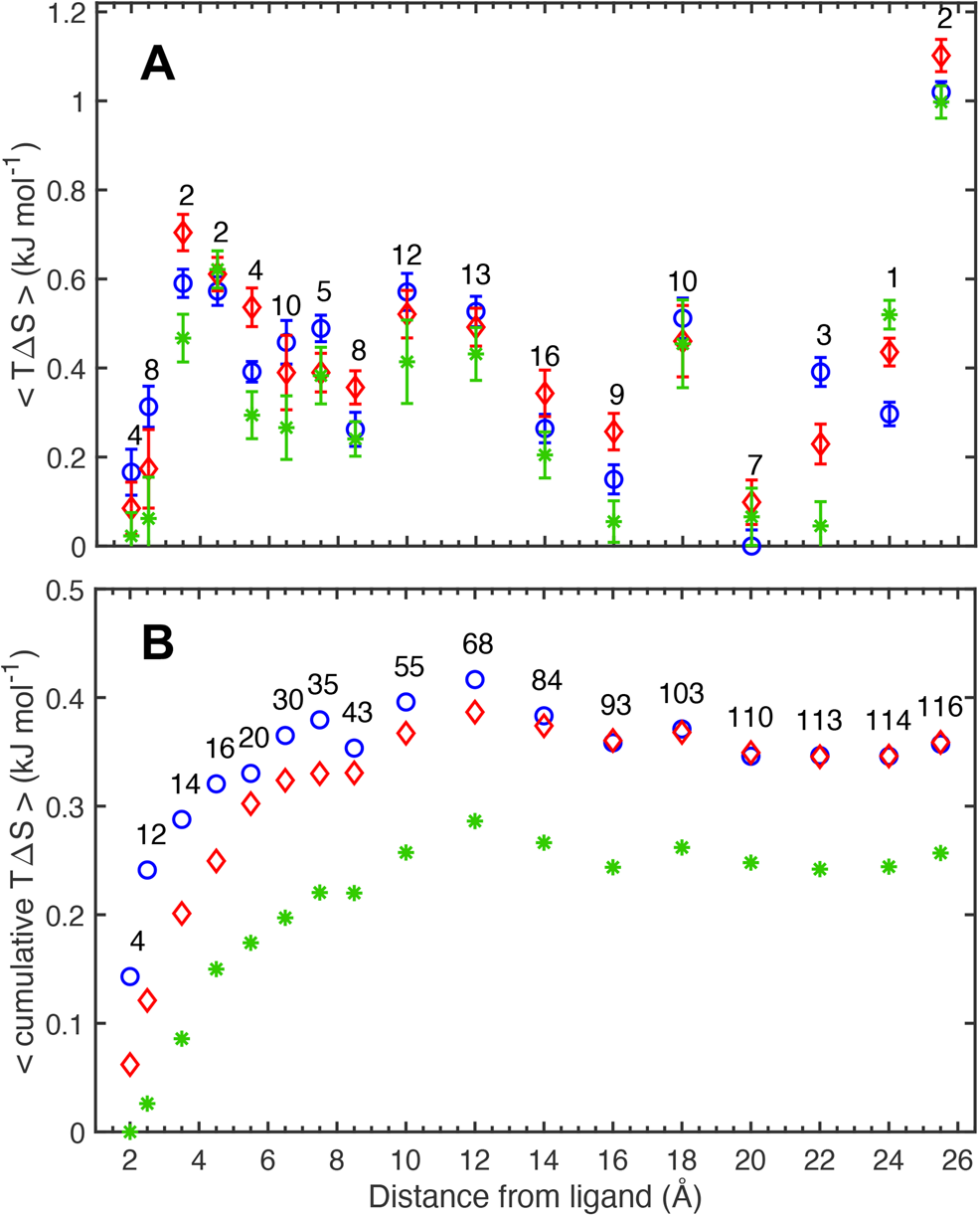
Radial distribution of the backbone conformational entropy in concentric shells around the binding site. The average backbone conformational entropy per residue *T*Δ*S*, estimated from NMR order parameters, is plotted as a function of the closest distance between any atom in the protein residue and the ligand. Entropies are referenced to the lowest entropy of any shell. (A) Average backbone conformational entropy per residue in each shell. (B) Cumulative conformational entropy per residue. Blue circles, M–galectin-3C; red diamonds, P–galectin-3C; green stars, O–galectin-3C. The number of residues included in each shell is stated above the symbols. The width of the shell is 1 Å between 2 and 9 Å, and 2 Å between 9 and 27 Å. The distance is evaluated at the midpoint of the shell. For clarity, error bars are only reported in panel A. Table S5 lists the residues included in each shell.

Although <*O*^2^> for the backbone is close to identical among the three complexes, the radial distribution of the backbone entropy (Figure 9) reveals differences in mobility for residues close to the binding site. Table S7 lists which residues belong to each shell. The NMR-based entropies reveal that the four residues of the innermost shell, His158, Arg162, Asn174, and Glu184, are among the most rigid in all complexes. These residues also form hydrogen bonds with the ligand (Figure 3). The next shell, 2–3 Å from the ligand, includes eight residues that are slightly less rigid. However, the following two shells (3–4 Å and 4–5 Å) comprise two residues each, whose flexibility is much higher than that for residues in the two innermost shells. The general pattern in Figure 9 reflects a dependence of secondary structure elements, with β-strands having low entropy, and loops and coils contributing to higher entropy; outside of the innermost shells, the proportion of residues in loops and coils is higher.

The distance dependence of the backbone entropy mirrors the greater backbone fluctuations in the binding sites of M– and P–galectin-3C compared to O–galectin-3C (cf., Figure 7). Since many residues have similar *O*^2^, the differences between the cumulative entropy curves are dominated by just a few residues (Figure 9B). In the innermost shell, the main difference is attributed to Arg162, with an *O*^2^ of 0.83, 0.85, and 0.87 in M–, P–, and O–galectin-3C. In the second shell (2–3 Å), Trp181, Arg186, Asn160, and Val172 show similar trends with *O*^2^ progressively increasing in the order M–, P–, and O–galectin-3C.

### Water Binding Sites and Solvation Thermodynamics

We identified water sites around the ligand-binding site and determined the solvent thermodynamics using GIST calculations based on MD simulations, in which the solute was restrained to the crystal structure.^77^ The results for the O, M, and P complexes reveal differences in their hydration thermodynamics, relative to bulk water (Table 3).

**Table 3:** Solvation Entropy from GIST Calculations^a^

complex	–*TΔS*_rot_	*–TΔS*_trans_	*–TΔS*_water_
M–galectin-3C	249.1 ± 0.1	183.3 ± 0.2	432.4 ± 0.3
P–galectin-3C	245.3 ± 0.2	183.0 ± 0.2	428.4 ± 0.3
O–galectin-3C	235.6 ± 0.2	166.5 ± 0.2	402.1 ± 0.3

a Units of kJ/mol; −*T*Δ*S*_rot_, solvent rotational entropy; −*T*Δ*S*_trans_, solvent translational entropy; −*T*Δ*S*_water_ = −*T*Δ*S*_rot_ – *T*Δ*S*_trans_. Errors are given as ±1 SD.

**Figure 10:**
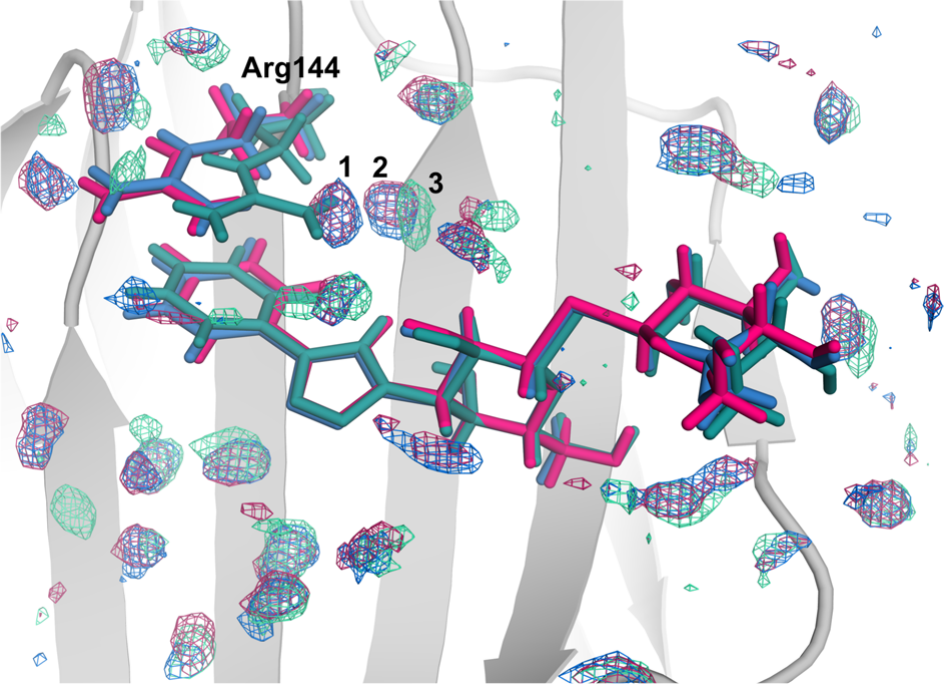
Differences in solvation around the binding site. Regions with a higher density of water molecules relative to bulk water (8 times the bulk water density) are represented as blue, red, and green meshes for M–, P–, and O–galectin-3C, respectively. Differences are observed nearby the side chain of Arg144, where water sites 1 and 2 are occupied in the M and P complexes, whereas in the O complex, only one water molecule is observed in site 3 because of the slightly different orientation of the Arg144 guanidino group.

M– and P–galectin-3C have similar solvation entropies, *–T*Δ*S*_water_, of 432.4 ± 0.3 and 428.4 ± 0.3 kJ/mol, respectively, whereas O–galectin-3C differs by having a less unfavorable solvation entropy of 402.1 ± 0.3 kJ/mol. Figure 10 shows an overview of the identified water sites, i.e., regions with higher density than bulk water, surrounding the bound ligands. Most water sites are highly similar among the three complexes, but a few distinct differences are observed. The less favorable solvation entropy of the M and P complexes primarily arises from the difference in the average conformation of Arg144 (cf., Figure 3B), which allows two water molecules to bridge between the protein and the ligand (positions 1 and 2, Figure 10), whereas in the O complex, the Arg144 side chain partly occupies this region of the binding site, leaving room for only one water molecule (position 3, Figure 10). These water molecules are observed also in the crystal structures (two for M and P but one for O, at positions within 0.5–0.6 Å of the centers of the GIST densities), although water molecule 2 is replaced by a chloride ion in the structures of the M and P complexes. The MD simulations indicate that these water-binding sites are highly dynamic with residence times for individual water molecules of only ∼0.3 ns for position 1 and 1–3 ns for positions 2 and 3. These residence times are in perfect agreement with experimental results obtained on lactose-bound galectin-3C.^13^

Solvation free energies of the free ligands in water solution, obtained by COSMO-RS calculations, are identical within errors (Table S5). Moreover, the total solvation entropies, calculated by GIST, for the free ligands agree within 0.7 kJ/mol (Table S5). Thus, the differences in total solvation entropy of binding among the three systems is governed by the observed differences in hydration of the complexes.

## Conclusions

**Figure 11:**
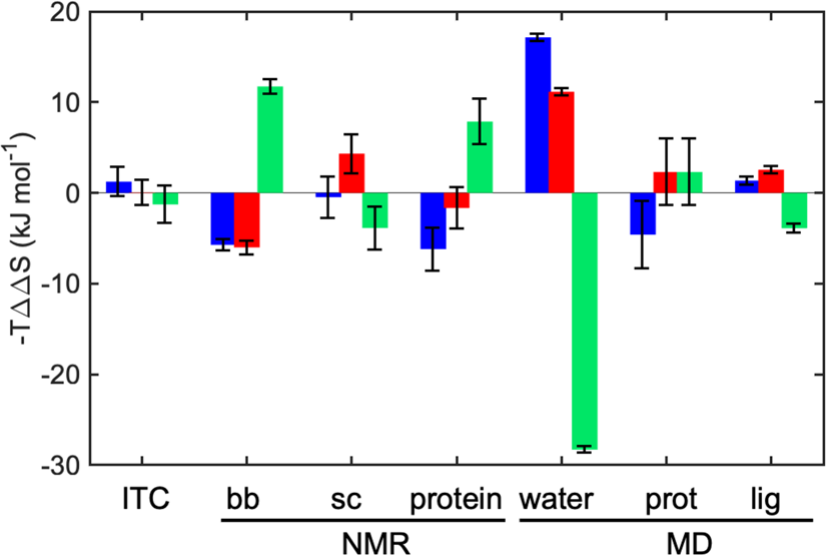
Entropy contributions to the differential binding of ligands M (blue), P (red), and O (green) to galectin-3C. The colored bars represent intercomplex differences in entropy; hence, a negative value corresponds to a favorable entropic contribution to binding for the specified complex, relative to the other two complexes. ITC reports the total entropy of binding, Table 1. NMR reports estimates of the conformational entropy of the backbone (bb) and methyl-bearing side chains (sc), Table 2. MD reports the conformational entropy of the protein (prot) and ligand (lig), Table S4, and the solvation entropy determined by GIST (water), Tables 3 and S5. Error bars indicate ±1 SD.

In this study, we compared the thermodynamic signature of binding three congeneric ligands, differing only in the position of a fluorine atom on a phenyl ring, to the carbohydrate binding domain of galectin-3. We used a multipronged approach to investigate in great detail the statistical thermodynamic driving forces for selectivity in binding the three ligands. Specifically, we break down the total entropy of binding into individual contributions from the different degrees of freedom, viz., protein, ligand, and solvent, as summarized in Figure 11.

The minor structural difference between the ligands renders their chemical potentials in the free state highly similar, as confirmed by calculations of conformational entropies, GIST solvation entropies, and COSMO-RS solvation free energies; the results do not differ, within the calculated uncertainties, between the three ligands (Table S5). Hence, the difference in binding thermodynamics can be related primarily to differences between the ligand–galectin-3C complexes. As might be expected from their structural similarity, the three ligands bind to galectin-3C with similar free energy. However, the O complex deviates from the other two in having less favorable binding enthalpy and less unfavorable binding entropy. The difference in binding enthalpy can ostensibly be rationalized in a straightforward manner by a reduced number of interactions involving the fluorine atom and surrounding protein in the O complex, compared to the M and P complexes. The difference in total entropy of binding arises as the net sum of opposing contributions from different components. The experimental results from NMR relaxation and ensemble-refined X-ray diffraction data suggest that the backbone of galectin-3C has reduced entropy when bound to O, especially for residues close to the binding site. (The MD-derived estimates of conformational entropy have large uncertainties and do not yield any significant differences between the three complexes.) One can speculate that the less favorable interactions of the *ortho*-fluorine atom with the protein create strain and restrict the flexibility of the protein backbone. However, this unfavorable entropic contribution to binding is apparently offset in the O complex, but not in the other two complexes, by increased entropy of the ligand and protein side-chains in immediate proximity with the ligand. The higher degree of flexibility of O can intuitively be associated with the fewer favorable protein–ligand interactions in this complex. Finally, the three complexes differ markedly in solvation entropy: A large difference in water entropy for the protein–ligand complexes favors the O complex.

Thus, the present results indicate an interesting case of *entropy–entropy compensation* between the conformational degrees of freedom of the protein, which disfavor binding of O over M or P, and the ligand and solvation entropies, which favor binding of O. These results highlight intricate details of binding thermodynamics and the importance of accounting for all components of the system in order to advance prospects for rational drug design.

## Supporting Information Available

Synthesis of ligands M and P, crystallography data collection and refinement statistics, electron density image for ligands, assigned ^1^H–^15^N and ^1^H–^13^C HSQC spectra, intercomplex chemical shift perturbations, ^15^N *R*_1_, *R*_2_, and {^1^H–^15^N}-NOE data together with back-calculated relaxation rates based on optimized model-free parameters, ^19^F *R*_2_ data, histograms of backbone and methyl *O*^2^ distributions, pairwise comparison of NMR order parameters for the complexes, comparison of backbone order parameters determined by MD and NMR, tables of backbone and methyl order parameters, table of conformational entropy differences between free and bound states determined by MD, table of conformational entropy and solvation free energy of the free ligands, table of results from MM/GBSA and interaction entropy (IE) calculations, methods section describing the MM/GBSA and IE calculations, table listing residues in each shell of the radial distribution of conformational entropy (PDF)

## Accession Codes

The crystal structures and the diffraction data have been deposited in the Protein Data Bank under accession IDs 6RZG (M complex), 6RZH (P complex), and 6RZF (O complex). The chemical shift assignments, relaxation rate constants, and order parameters have been deposited in the Biological Magnetic Resonance Bank (BMRB) under accession codes 50283 (M complex), 50284 (P complex), and 50285 (O complex).
