# Supplementary material for: Entropy–Entropy Compensation between the Protein, Ligand, and Solvent Degrees of Freedom Fine-Tunes Affinity in Ligand Binding to Galectin-3C
Source: JACS Au. 2021 Apr 1;1(4):484–500. doi: 10.1021/jacsau.0c00094 (PMC8395690; doi:10.1021/jacsau.0c00094)
Supplement: Supplementary file 1 — au0c00094_si_001.pdf [file au0c00094_si_001.pdf]

Supporting Information

Entropy–Entropy Compensation Between the Protein, Ligand, and Solvent Degrees of Freedom Fine-tunes Affinity in Ligand Binding to Galectin-3C

Johan Wallerstein[1], Vilhelm Ekberg[2‡], Majda Misini Ignjatović[2‡], Rohit Kumar[3‡], Octav Caldararu[2], Kristoffer Peterson[4], Sven Wernersson[1], Ulrika Brath[5], Hakon Leffler[6], Esko Oksanen[7], Derek T. Logan[3], Ulf J. Nilsson[4], Ulf Ryde[2,*], and Mikael Akke[1,*]

[1]Biophysical Chemistry, Center for Molecular Protein Science, Department of Chemistry, Lund University, 221 00 Lund, Sweden; [2]Theoretical Chemistry, Department of Chemistry, Lund University, 221 00 Lund, Sweden; [3]Biochemistry and Structural Biology, Center for Molecular Protein Science, Department of Chemistry, Lund University, 221 00 Lund, Sweden; [4]Centre for Analysis and Synthesis, Department of Chemistry, Lund University, 221 00 Lund, Sweden; [5]The Swedish NMR Center, University of Gothenburg, 405 30 Gothenburg, Sweden; [6]Microbiology, Immunology, and Glycobiology, Department of Experimental Medicine, Lund University, 221 00 Lund, Sweden; [7]European Spallation Source ESS ERIC, 225 92 Lund, Sweden

* corresponding authors: ulf.ryde@teokem.lu.se; mikael.akke@bpc.lu.se

SYNTHESIS OF LIGANDS M AND P

Materials and methods

Purification of compounds was carried out by preparative HPLC (Agilent 1260 infinity system, column SymmetryPrep-C18, 17 ml/min H2O-MeCN gradient 10–100% 15 min with 0.1% formic acid acid). Specific rotations were measured on a Perkin Elmer model 341 polarimeter. NMR spectra ^{1}H, ^{13}C, 2D COSY and HMQC were recorded with a Bruker Avance II 400 MHz spectrometer at ambient temperature. Chemical shifts are reported in δ parts per million (ppm). HRMS was determined by direct infusion on a Waters XEVO-G2 QTOF mass spectrometer using electrospray ionization (ESI). Ligands **M** and **P** were of >95% purity according to UPLC (Waters Acquity UPLC system, column Waters Acquity CSH C18, 0.5 ml/min H_2O-MeCN gradient 5–95% 10 min with 0.1% formic acid) analysis.

General procedures for preparation of (M) and (P)

To a solution of 3'-azido-3'-deoxy-β-D-galactopyranosyl 1-thio-β-D-glucopyranoside (20 mg, 0.052 mmol) and cupper iodide (2.5 mg, 0.013 mmol) in acetonitrile (3 mL) were the corresponding acetylene derivative (x, 1.5 equiv) and diisopropylethylamine (9 µL, 0.052 mmol) added. The mixture was stirred for 24 h at 50 °C before quenching with sat. aq. NH_4Cl followed by evaporation of the solvent. The residue was purified with preparative HPLC to give the product as an amorphous white solid.

3'-[4-(3-fluorophenyl)-1*H*-1,2,3-triazol-1-yl]-3'-deoxy-β-D-galactopyranosyl 1-thio-β-D-glucopyranoside (M)

x=3-fluorophenylacetylene. Yield 12 mg, 46%. $[\alpha]_D^{20}$ 21.5 (c 0.87, CH_3OH). ^{1}H NMR (CD_3OD, 400 MHz): δ 8.48 (s, 1H, Ph), 7.66 (td, *J* = 7.8, 1.2 Hz, 1H, Ph), 7.60 (ddd, *J* = 10.1, 2.5, 1.5 Hz, 1H, Ph), 7.45 (td, *J* = 8.0, 6.0 Hz, 1H, Ph), 7.08 (ddt, *J* = 8.8, 2.6, 0.9 Hz, 1H, Ph), 4.94 (d, *J* = 9.6 Hz, 1H, H-1), 4.87 (obscured by water H-3), 4.79 (d, *J* = 9.8 Hz, 1H, H-1'), 4.33 (t, *J* = 10.1 Hz, 1H, H-2), 4.13 (d, *J* = 2.8 Hz, 1H, H-4), 3.90 (dd, *J* = 12.2, 1.7 Hz, 1H, H-6'), 3.86–3.78 (m, 2H, H-5 and H-6), 3.71–3.64 (m, 2H, H-6 and H-6'), 3.43–3.32 (m, 4H, H-2', H-3', H-4' and H-5'). ^{13}C NMR (CD_3OD, 100 MHz): δ 165.9, 163.5, 147.2, 134.3, 131.9, 122.4, 115.7, 113.2, 86.0, 84.5, 82.2, 81.2, 79.6, 74.7, 71.5, 69.8, 69.2, 68.5, 63.0, 62.6. HRMS calculated for $[C_{20}H_{27}FN_3O_9S]^+$, 504.1452; found: 504.1451.

3'-[4-(4-fluorophenyl)-1*H*-1,2,3-triazol-1-yl]-3'-deoxy-β-D-galactopyranosyl 1-thio-β-D-glucopyranoside (P)

x=4-fluorophenylacetylene. Yield 10 mg, 38%. $[\alpha]_D^{20}$ 16.3 (c 0.83, CH_3OH). ^{1}H NMR (CD_3OD, 400 MHz): δ 8.41 (s, 1H, Ph), 7.86 (dd, *J* = 8.9, 5.3 Hz, 2H, Ph), 7.18 (t, *J* = 8.9 Hz, 2H, Ph), 4.94 (d, *J* = 9.6 Hz, 1H, H-1), 4.87 (obscured by water H-3), 4.79 (d, *J* = 9.8 Hz, 1H, H-1'), 4.33 (t, *J* = 10.1 Hz, 1H, H-2), 4.13 (d, *J* = 2.8 Hz, 1H, H-4), 3.90 (dd, *J* = 12.2, 1.7 Hz, 1H, H-6'), 3.86-3.78 (m, 2H, H-5 and H-6), 3.71-3.64 (m, 2H, H-6 and H-6'), 3.43-3.32 (m, 4H, H-2', H-3', H-4' and H-5'). ^{13}C NMR (CD_3OD, 100 MHz): δ 165.3, 162.9, 147.4, 128.6, 121.7, 116.8, 86.0, 84.5, 82.2, 81.2, 79.6, 74.7, 71.5, 69.8, 69.1, 68.5, 63.0, 62.6. HRMS calculated for $[C_{20}H_{27}FN_3O_9S]^+$, 504.1452; found: 504.1452.

Table S1 Data collection and refinement statistics for analysis of X-ray crystallography data

compound	***meta***	***para***	***ortho***
**PDB code**	**6RZG**	**6RZH**	**6RZF**
**station**	P13(EMBL)	P13(EMBL)	P13(EMBL)
**wavelength [Å]**	0.9299	0.9299	0.9299
**unit cell (Å)**	a = 36.30<br>b = 57.98<br>c = 62.52	a =36.16<br>b = 57.88<br>c = 62.45	a = 36.27<br>b = 57.62<br>c = 62.01
**space group**	$P2_12_12_1$	$P2_12_12_1$	$P2_12_12_1$
**resolution range [Å]**	31.26 - 1.01<br>(1.05 - 1.015)	31.29 - 0.94<br>(0.98 - 0.94)	31.01-1.01<br>(1.05 - 1.01)
**completeness [%]**	99.4 (94.7)	98.9 (89.7)	98.0 (86.9)
**Total reflections**<br>**unique reflections**	833346 (65896)<br>68826 (6735)	987114(67628)<br>83126 (7464)	789647 (55524)<br>66460 (6016)
**CC1/2**	1.000 (0.254)	0.999 (0.484)	1.000 (0.387)
**multiplicity**	12.1 (9.8)	11.9 (9.1)	11.9 (9.2)
**R_{merge} [%]**	0.066 (2.887)	0.052 (1.485)	0.067 (2.043)
**mean I/σ(I)**	15.5 (0.5)	18.3 (1.0)	14.9 (0.7)
**Wilson B-factor [Å^2]**	11.6	10.3	11.3
**R_{model} (F) [%]**	0.139 (0.317)	0.132 (0.279)	0.137 (0.315)
**R_{free} (F) [%]**	0.154 (0.331)	0.144 (0.291)	0.159 (0.335)
**reflections used in refinement**<br>**(for R_{free})**	68536 (6451)<br>3200 (313)	83084 (7428)<br>4200 (355)	66243 (5803)<br>3471 (315)
**average B-factors [Å^2]**	protein: 13.7<br>ligand: 15.9<br>solvent: 28.3	protein: 12.4<br>ligand: 14.0<br>solvent: 27.2	protein: 13.1<br>ligand:19.7<br>solvent: 26.3
**Ramachandran outliers [%]**	0.0	0.0	0.00
**rotamer outliers [%]**<br>**MolProbity clash score**	0.0<br>1.22	1.48<br>2.06	0.76<br>0.85
**bond length rmsd from ideal [Å]**<br>**bond angle rmsd from ideal [°]**	0.012<br>1.32	0.012<br>1.25	0.012<br>1.28

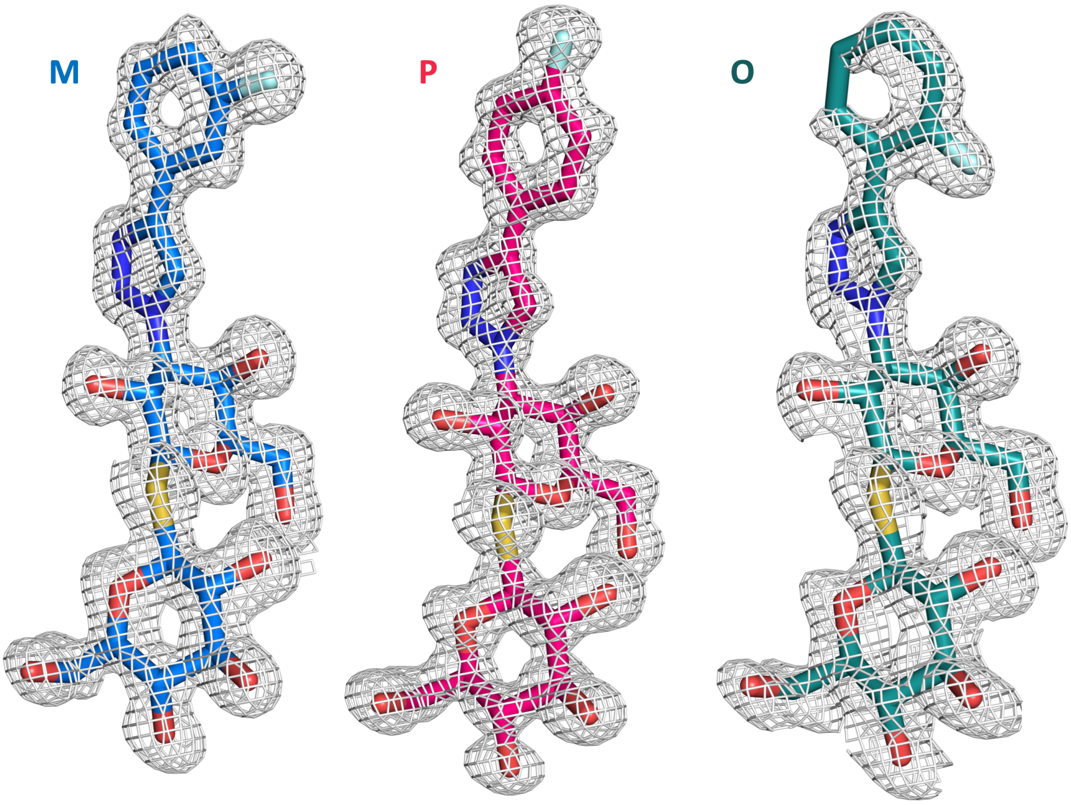

Figure S1. Unambiguous electron density for the ligands M, P and O, represented by an isomesh view.

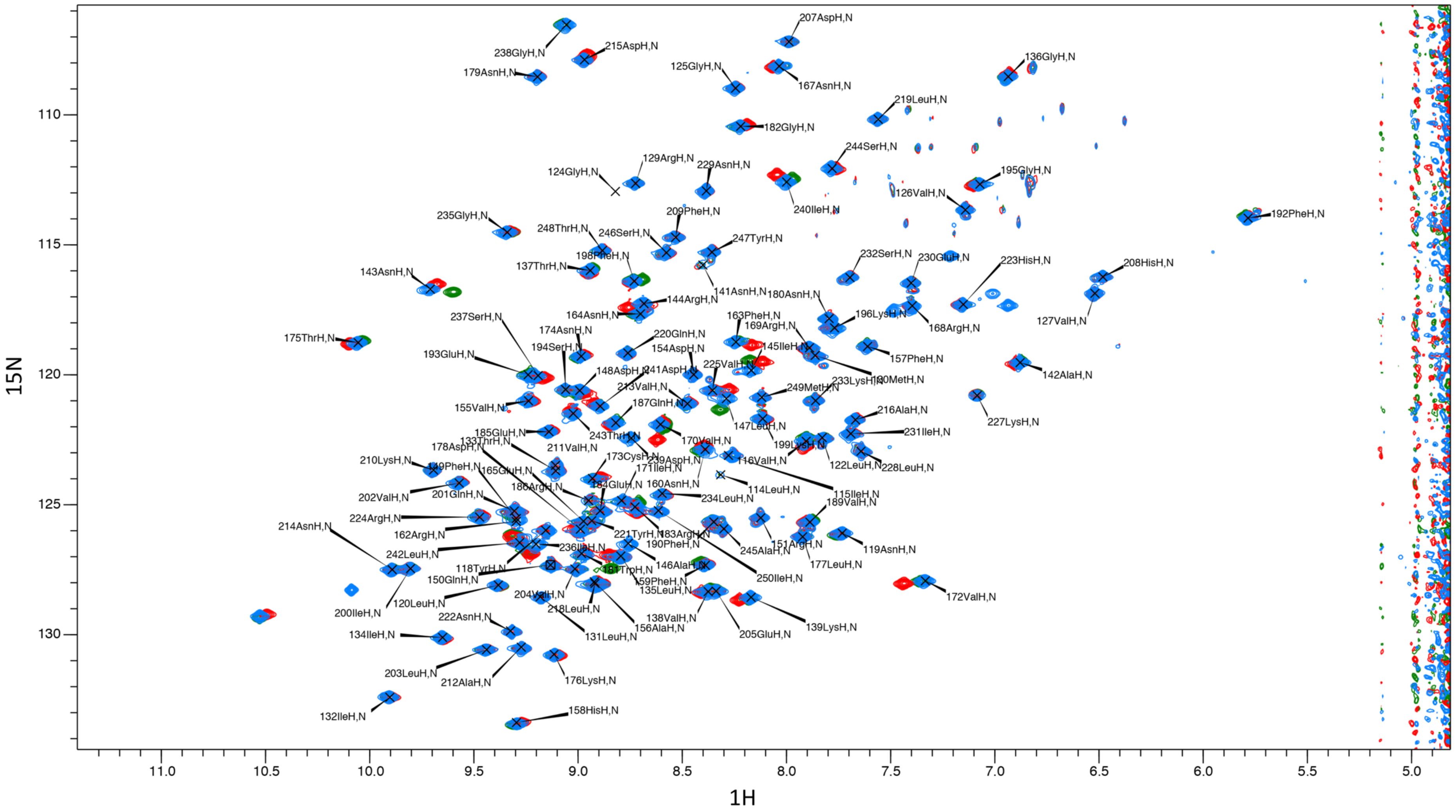

Figure S2. ^{15}N–^{1}H HSQC correlation spectra of M–, P– and O–galectin-3C in blue, red and green, respectively, acquired at 14.1 T and 301K.

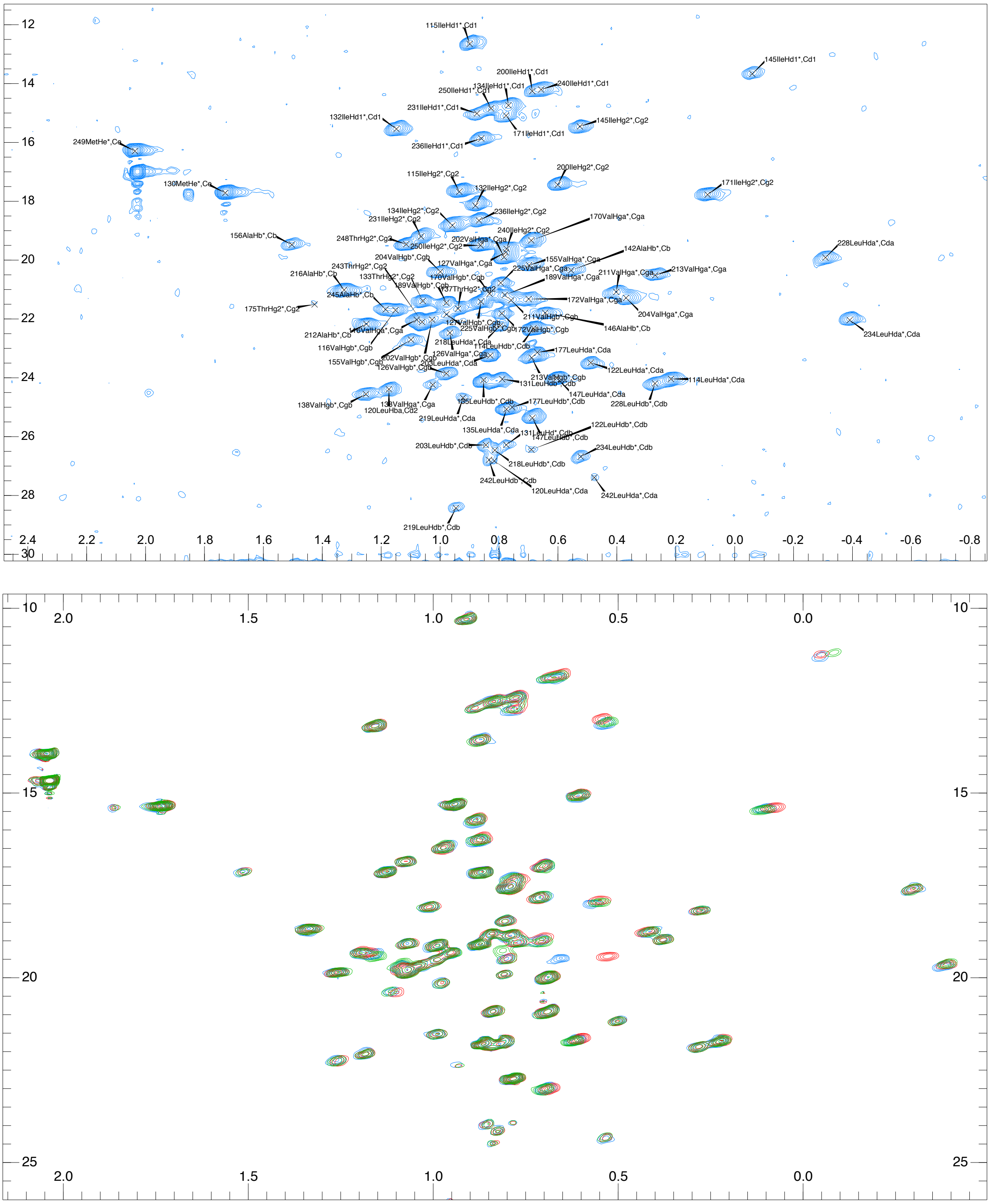

Figure S3. ^{1}H-^{13}C HSQC correlation spectra of the methyl region of M–, P– and O–galectin-3C acquired at 14.1 T and 301 K. The upper panel shows the assigned spectrum for M–galectin-3C. The lower panel shows the superimposed spectra of M–, P– and O–galectin-3C in blue, red and green, respectively. The horizontal and vertical axes show the ^{1}H and ^{13}C chemical shifts, respectively.

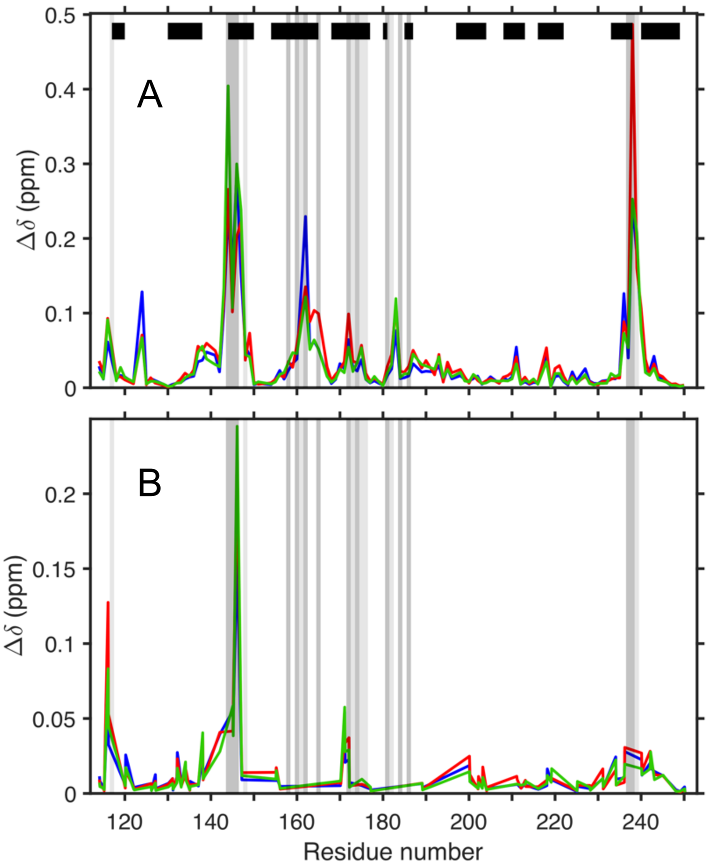

Figure S4. Chemical shift perturbations upon ligand binding. (A) ^{1}H-^{15}N backbone chemical shift differences. (B) ^{1}H-^{13}C side chain methyl chemical shift differences. $\Delta\delta$ refers to the inter-complex comparison of chemical shifts calculated as the sum of vector norms, $\Delta\delta_A = (\Delta\delta_{AB} + \Delta\delta_{AC})/2$, with $\Delta\delta_{AB} = ([\Delta\delta_{AB}(^{1}H)]^2 + [0.16\Delta\delta_{AB}(^{15}N)]^2)^{1/2}$ in the case of the backbone chemical shift differences, or $\Delta\delta_{AB} = ([\Delta\delta_{AB}(^{1}H)]^2 + [0.25\Delta\delta_{AB}(^{13}C)]^2)^{1/2}$ for the methyl chemical shift differences, etc. Blue, red, and green represent M–, P–, and O–galectin-3, respectively. Panel A includes data for 122 amide backbone peaks, while panel B includes 85 methyl side chain peaks. Black horizontal bars represent the location of the β-sheet secondary structure elements. The vertical bars indicate residues close to binding pocket: dark (light) grey indicates residues within 4 Å (6 Å) from any ligand atom.

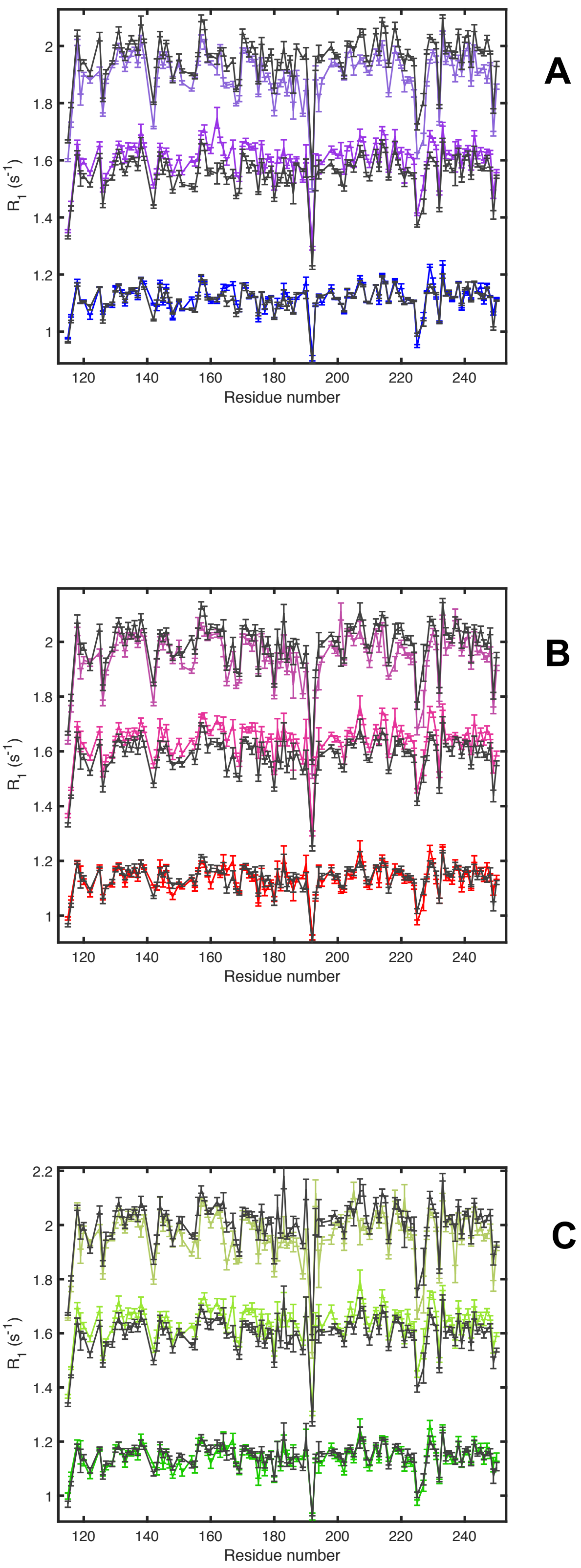

Figure S5. ^{15}N R_1 relaxation rates included in the model-free analysis. The colored symbols represent experimental data, whereas the black symbols indicate the values back-calculated from the fitted model-free parameters. M–, P– and O–galectin-3 are shown in blue (A), red (B) and green (C), respectively. In each panel the data are ordered according to the magnetic field strength, from bottom to top: 18.8 T, 14.1 T and 11.7 T.

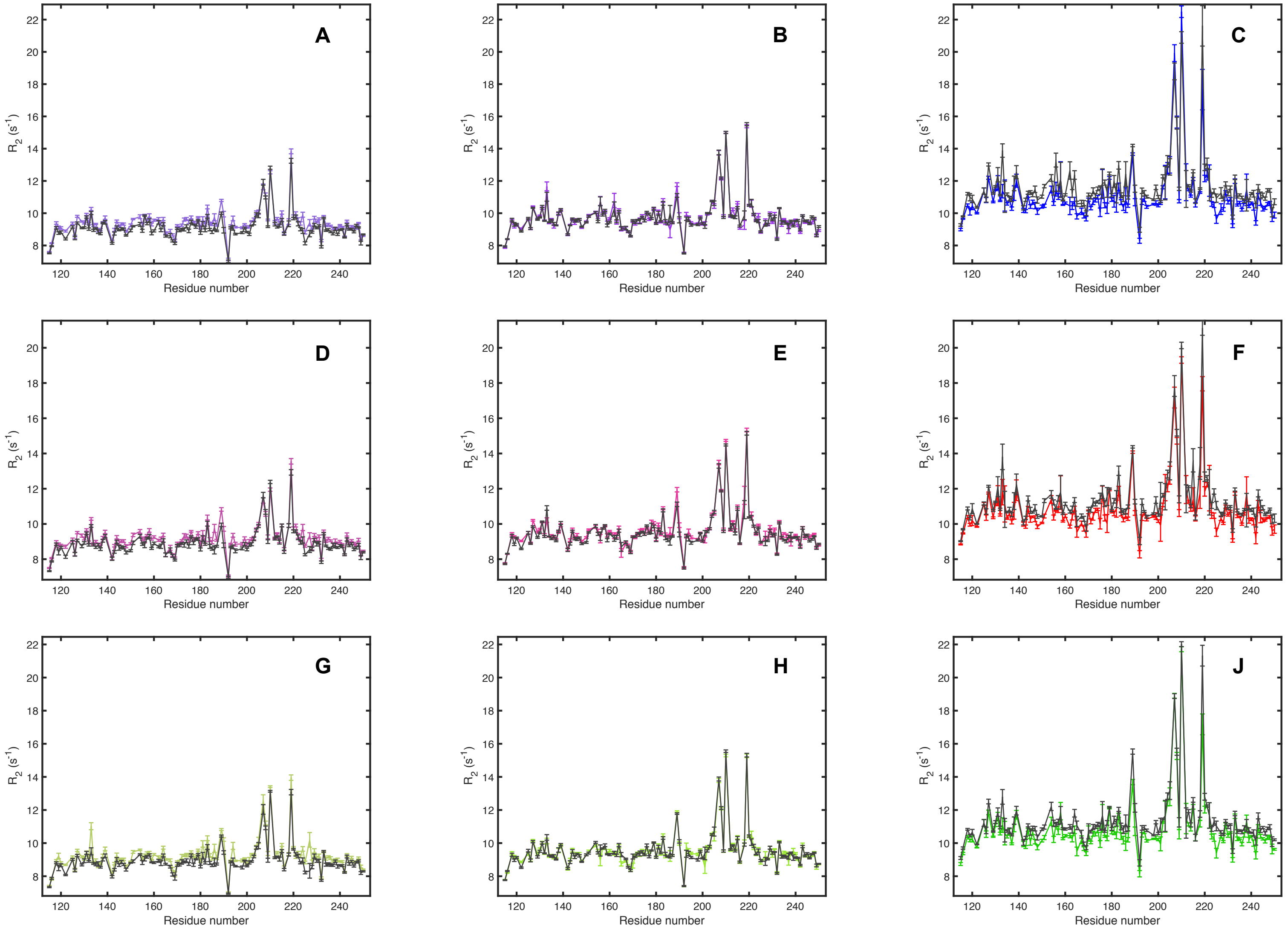

Figure S6. ^{15}N R_2 relaxation rates included in the model-free analysis. The colored symbols represent experimental data, whereas the black symbols indicate the values back-calculated from the fitted model-free parameters. (A–C) M–galectin-3, (D–F) P–galectin-3, (G–J) O–galectin-3. The magnetic field strength is from left to right: 11.7 T, 14.1 T and 18.8 T.

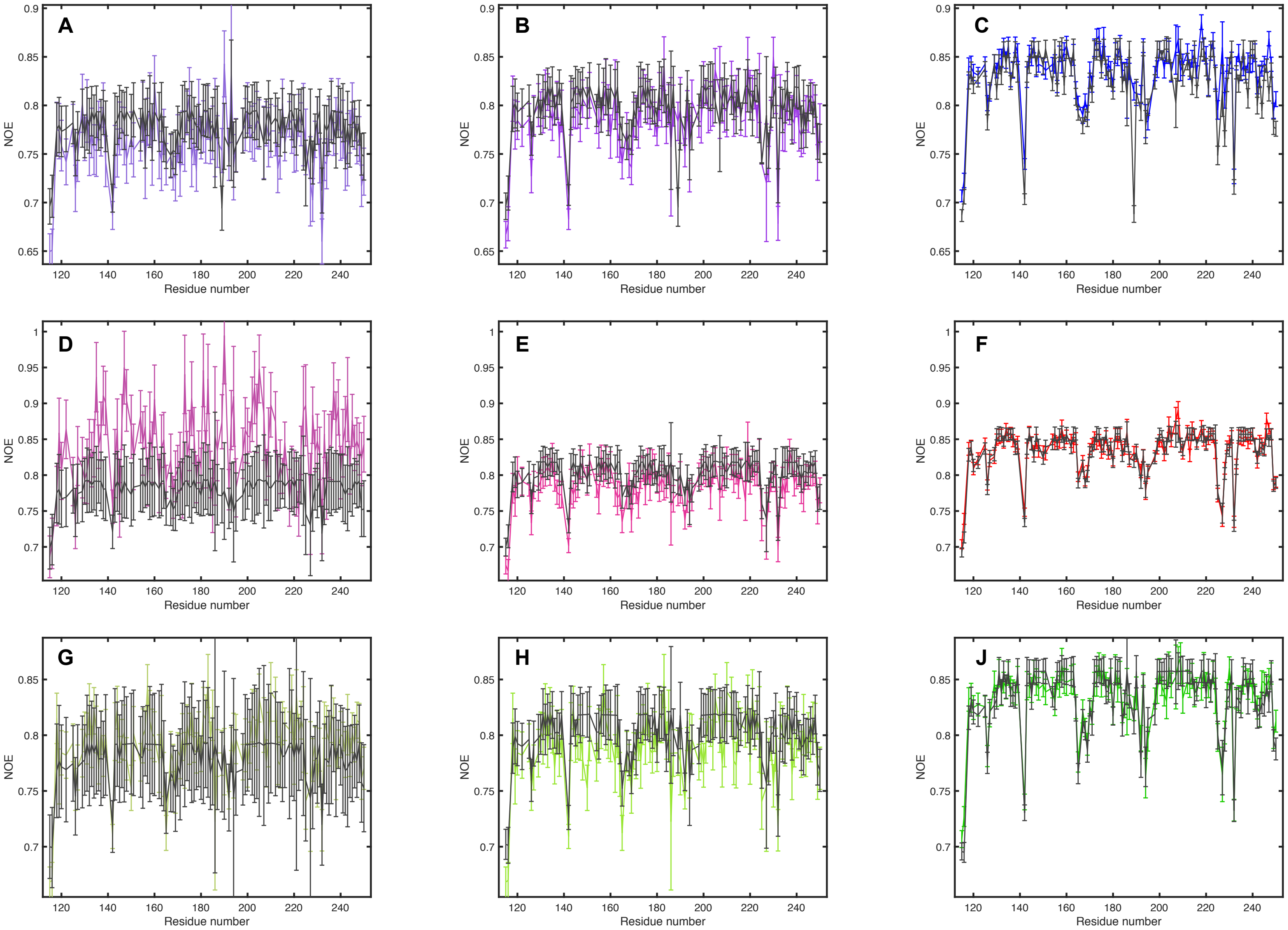

Figure S7. {^{1}H}-^{15}N *NOE* values included in the model-free analysis. The colored symbols represent experimental data, whereas the black symbols indicate the values back-calculated from the fitted model-free parameters. (A–C) M–galectin-3, (D–F) P–galectin-3, (G–J) O–galectin-3. The magnetic field strength is from left to right: 11.7 T, 14.1 T and 18.8 T.

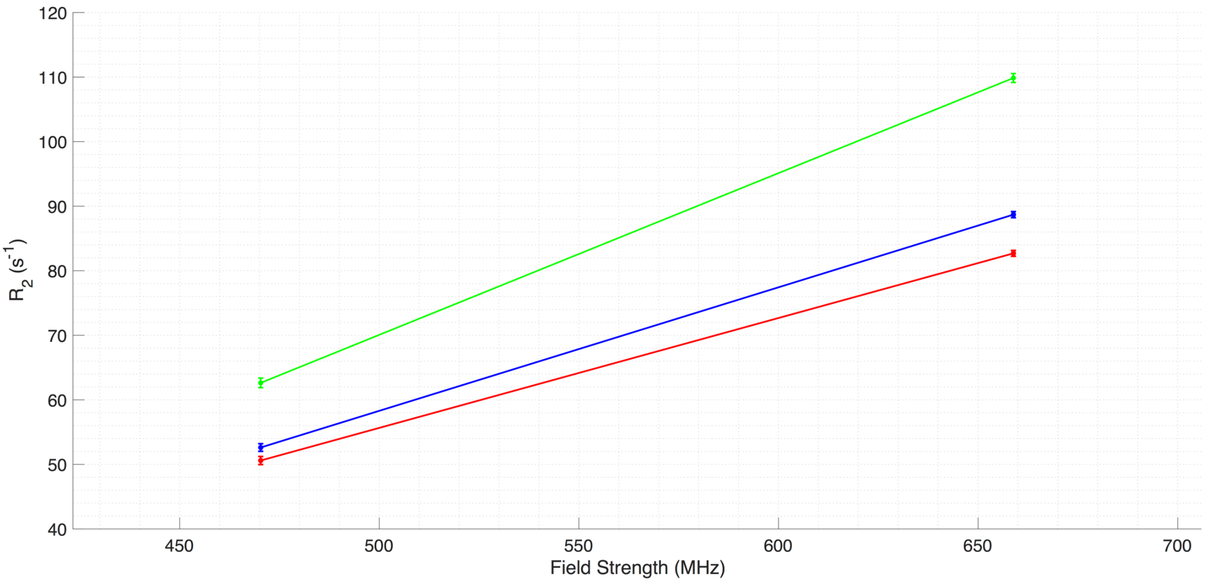

Figure S8. ^{19}F R_2 relaxation rates probing the dynamics of the bound ligands. Blue, red, and green represent M–, P–, and O–galectin-3, respectively.

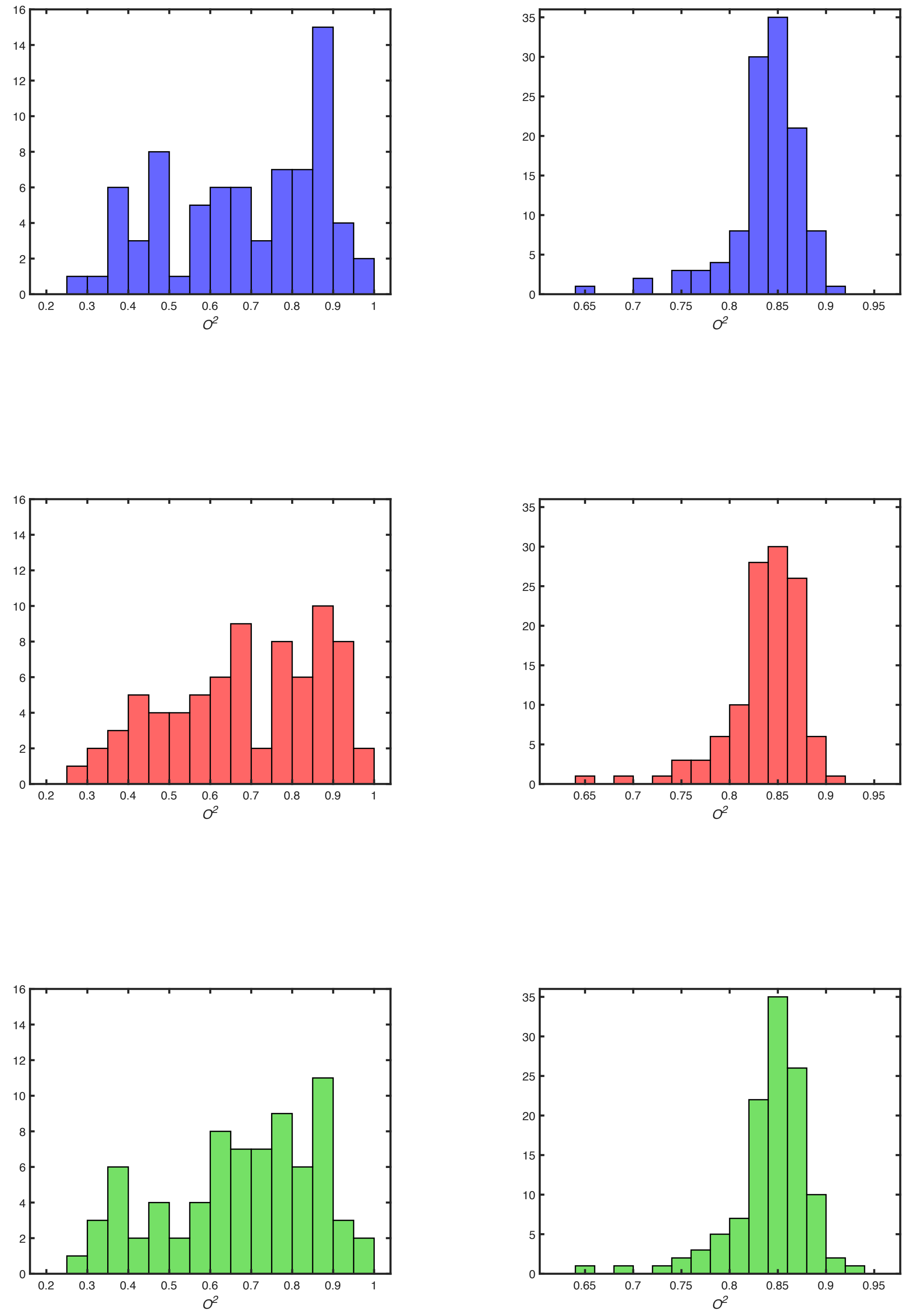

Figure S9. Histogram of order parameters O^2 for M–, P– and O–galectin-3 in blue, red and green respectively. The left-hand column shows the methyl side chain O^2 (bin size 0.05) and right-hand column shows the backbone amide O^2 (bin size 0.02).

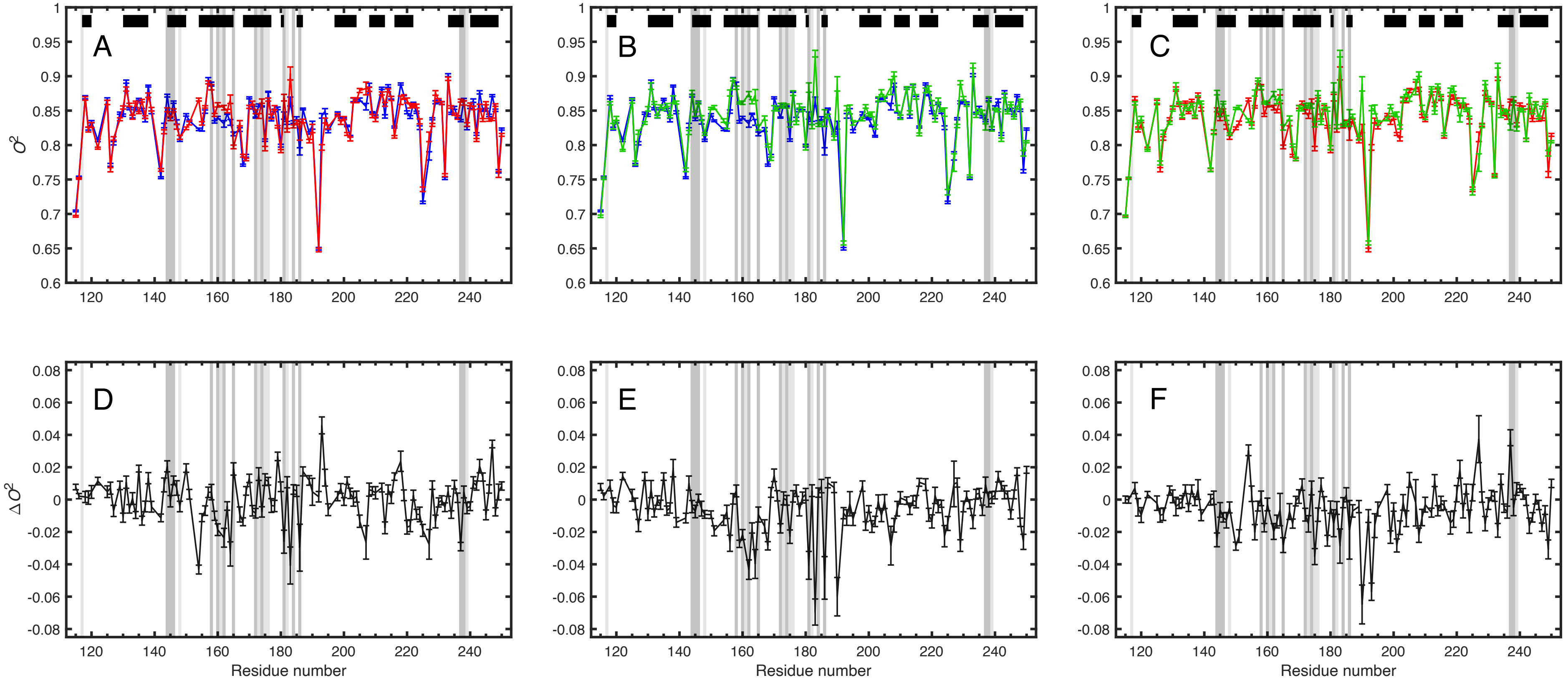

Figure S10. Pairwise comparisons of backbone order parameters (A–C). Blue, red, and green represent M–, P–, and O–galectin-3, respectively. The corresponding differences are shown in the second row of panels: M–P (D); M–O (E); and P–O (F). Error bars indicate 1 SD. Black horizontal bars represent the location of the β-sheet secondary structure elements. The vertical bars indicate residues close to binding pocket: dark (light) grey indicates residues within 4 Å (6 Å) from any ligand atom. A two-sample *t*-test was performed testing the null hypothesis of equal mean values, $\langle O^2 \rangle$. The resulting *p*-values are 0.97 for M vs P, 0.26 for M vs O, and 0.25 for P vs O; hence none of the *t*-tests rejects the null hypothesis, and the means are assumed to be equal. This result is in line with that of the inter-complex comparisons reported in the main text.

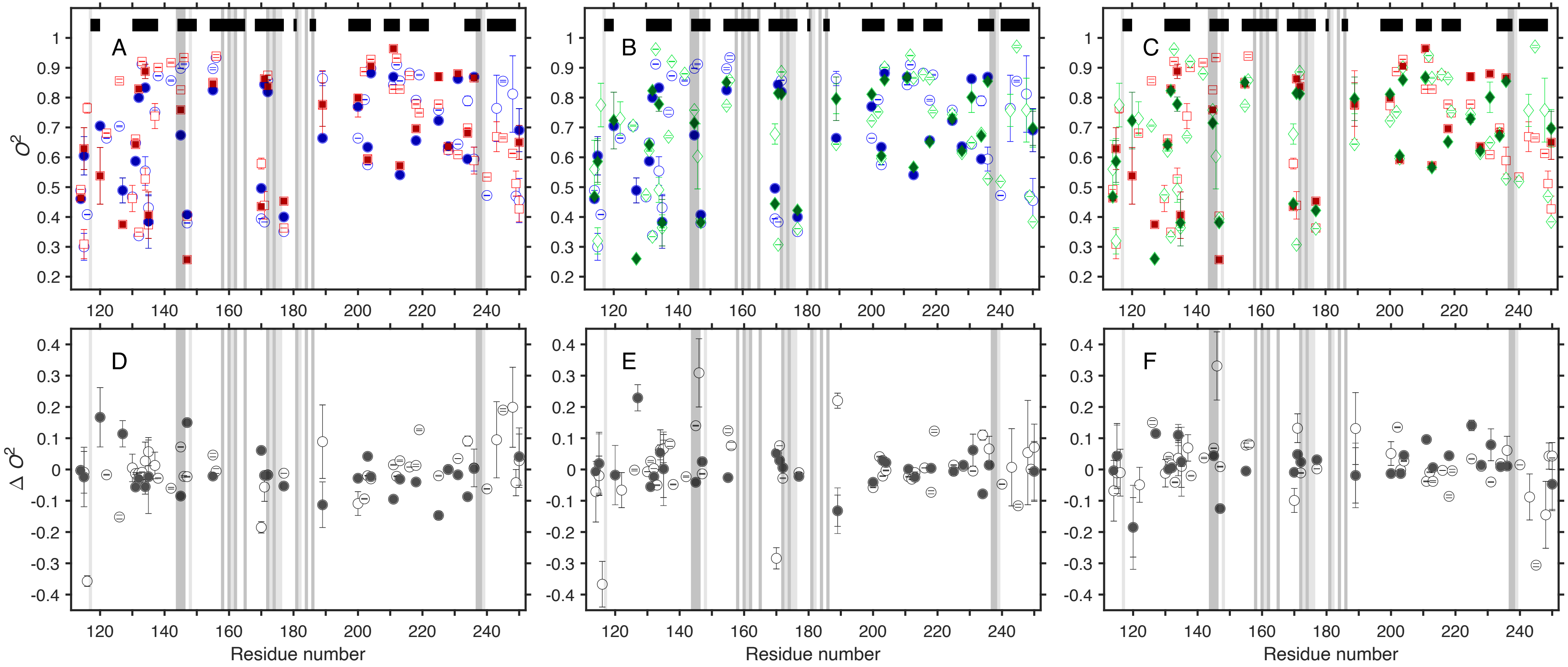

Figure S11. Pairwise comparisons of methyl order parameters (A–C). Blue, red, and green represent M–, P–, and O–galectin-3, respectively. The corresponding differences are shown in the second row of panels: M–P (D); M–O (E); and P–O (F). Error bars indicate 1 SD. Open markers represent residues with a single methyl group (Ala, Thr, and Met) and methyl groups Cγ1 (Val), Cδ1 (Leu) or Cδ1 (Ile), whereas filled markers refer to Cγ2 (Val), Cδ2 (Leu) or Cγ2 (Ile). Error bars indicate 1 SD. Black horizontal bars represent the location of the β-sheet secondary structure elements. The vertical bars indicate residues close to binding pocket: dark (light) grey indicates residues within 4 Å (6 Å) from any ligand atom. A two-sample *t*-test was performed testing the null hypothesis of equal mean values, $<O^2>$. The *p*-values are 0.78 for M vs P, 0.86 for M vs O and 0.65 for P vs O; hence none of the *t*-tests rejects the null hypothesis, and the means are assumed to be equal. This result is in line with that of the inter-complex comparisons reported in the main text.

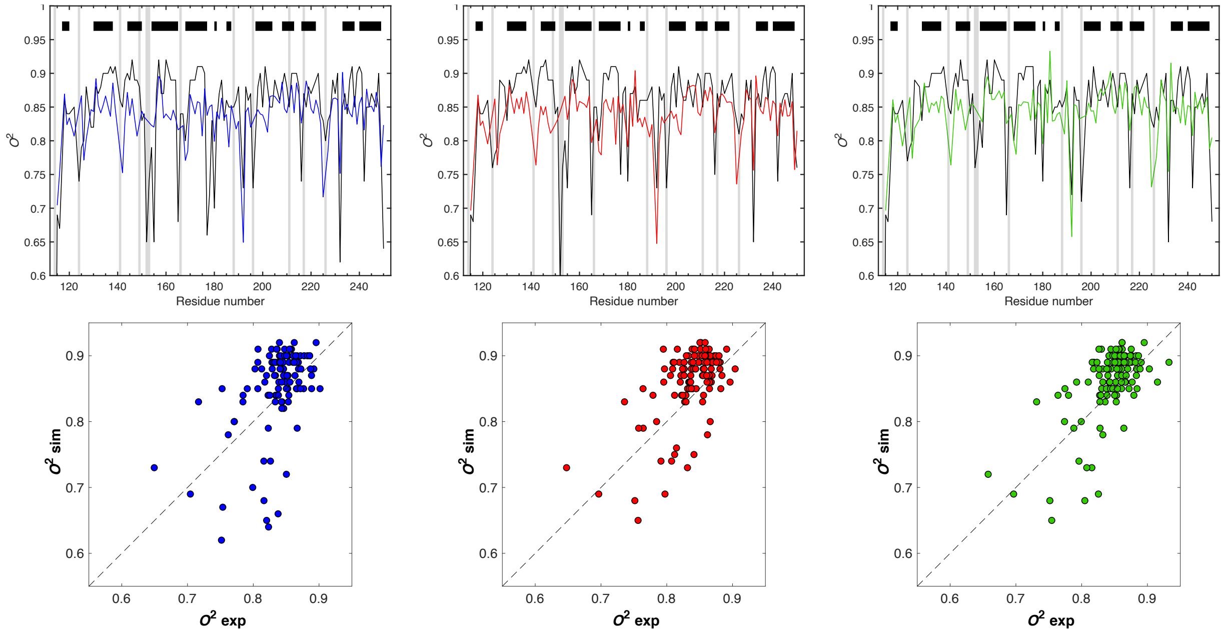

Figure S12. Pairwise comparisons of experimental and simulated backbone order parameters O^2 for the three complexes. Experimental O^2 are shown in color and simulated O^2 in black. Blue (left), red (middle), green (right) panels represent M–, P–, and O–galectin-3C, respectively. The top row of panels shows the backbone O^2. The experimental data comprise the same 116 residues as in Fig. 6 of the main text, while the simulated data comprise all 128 residues. The experimental data points are joined by continuous line segments although some data are not included; gray vertical bars indicate those residues for which experimental values are not available. The black horizontal bars indicate the location of the β-sheet secondary structure elements. The bottom row of panels shows correlation plots for the 116 residues included in the experimental data sets. The simulated O^2 are similar on average with $\langle O^2\rangle_{MD} - \langle O^2\rangle_{NMR}$ = 0.006, 0.013 and 0.007 for the M-, P- and O-complexes, respectively. The RMSE is 0.056, 0.044 and 0.043 for the three data sets.

Table S2. ^{15}**N backbone order parameters (*O*^{2}) with 1 SD errors for the three galectin-3C complexes**

Residue	Type	Meta		Para		Ortho	
		O^2	SD	*O*^{2}	SD	*O*^{2}	SD
115	ILE	0.7043	0.0012	0.6966	0.0012	0.6966	0.0018
116	VAL	0.7536	0.0011	0.7515	0.0011	0.7517	0.0017
118	TYR	0.8694	0.0023	0.8677	0.0025	0.8606	0.0023
119	ASN	0.8238	0.0021	0.8226	0.0036	0.8273	0.0041
120	LEU	0.8339	0.0017	0.8291	0.0036	0.8387	0.0029
122	LEU	0.8069	0.0018	0.7954	0.0014	0.7923	0.0013
125	GLY	0.8668	0.0018	0.8619	0.0031	0.8641	0.0032
126	VAL	0.7713	0.0021	0.7642	0.0031	0.7740	0.0029
127	VAL	0.8026	0.0024	0.8096	0.0025	0.8186	0.0029
129	ARG	0.8463	0.0016	0.8375	0.0021	0.8320	0.0020
130	MET	0.8434	0.0017	0.8546	0.0022	0.8526	0.0032
131	LEU	0.8921	0.0023	0.8813	0.0030	0.8856	0.0048
132	ILE	0.8528	0.0014	0.8585	0.0022	0.8611	0.0020
133	THR	0.8413	0.0020	0.8406	0.0031	0.8419	0.0021
134	ILE	0.8528	0.0016	0.8623	0.0014	0.8560	0.0023
135	LEU	0.8664	0.0014	0.8473	0.0016	0.8522	0.0019
136	GLY	0.8514	0.0020	0.8644	0.0019	0.8582	0.0020
137	THR	0.8363	0.0026	0.8406	0.0021	0.8415	0.0018
138	VAL	0.8857	0.0013	0.8724	0.0032	0.8662	0.0052
139	LYS	0.8476	0.0021	0.8520	0.0026	0.8617	0.0021
142	ALA	0.7526	0.0013	0.7639	0.0020	0.7641	0.0027
143	ASN	0.8284	0.0032	0.8193	0.0028	0.8182	0.0030
144	ARG	0.8718	0.0020	0.8512	0.0028	0.8761	0.0032
145	ILE	0.8375	0.0019	0.8385	0.0030	0.8449	0.0034
146	ALA	0.8623	0.0016	0.8504	0.0021	0.8615	0.0019
147	LEU	0.8336	0.0030	0.8264	0.0033	0.8392	0.0034
148	ASP	0.8069	0.0016	0.8112	0.0029	0.8161	0.0018
150	GLN	0.8438	0.0017	0.8243	0.0020	0.8532	0.0015
151	ARG	0.8352	0.0013	0.8320	0.0027	0.8545	0.0028
154	ASP	0.8228	0.0018	0.8661	0.0021	0.8356	0.0026
155	VAL	0.8203	0.0001	0.8315	0.0024	0.8279	0.0041
156	ALA	0.8486	0.0024	0.8554	0.0017	0.8756	0.0044
157	PHE	0.8955	0.0024	0.8911	0.0021	0.8951	0.0021
158	HIS	0.8885	0.0028	0.8839	0.0039	0.8832	0.0023
159	PHE	0.8337	0.0018	0.8437	0.0022	0.8611	0.0014
160	ASN	0.8398	0.0019	0.8584	0.0031	0.8614	0.0024
162	ARG	0.8307	0.0048	0.8543	0.0033	0.8740	0.0036

163	PHE	0.8454	0.0008	0.8494	0.0025	0.8630	0.0031
164	ASN	0.8329	0.0042	0.8665	0.0062	0.8727	0.0079
165	GLU	0.8162	0.0027	0.7973	0.0026	0.8255	0.0037
167	ASN	0.8225	0.0027	0.8317	0.0038	0.8391	0.0031
168	ARG	0.7708	0.0015	0.7845	0.0019	0.7994	0.0021
169	ARG	0.7845	0.0019	0.7791	0.0018	0.7800	0.0023
170	VAL	0.8685	0.0014	0.8554	0.0021	0.8536	0.0036
171	ILE	0.8544	0.0020	0.8613	0.0025	0.8521	0.0025
172	VAL	0.8400	0.0008	0.8484	0.0028	0.8577	0.0027
173	CYS	0.8580	0.0017	0.8423	0.0035	0.8573	0.0036
174	ASN	0.8562	0.0007	0.8638	0.0031	0.8566	0.0029
175	THR	0.8072	0.0017	0.7948	0.0032	0.8301	0.0038
176	LYS	0.8774	0.0007	0.8661	0.0023	0.8754	0.0034
177	LEU	0.8377	0.0026	0.8414	0.0034	0.8322	0.0024
178	ASP	0.8504	0.0018	0.8543	0.0030	0.8568	0.0031
179	ASN	0.8533	0.0019	0.8266	0.0018	0.8520	0.0028
180	ASN	0.7992	0.0020	0.7912	0.0022	0.7959	0.0031
181	TRP	0.8428	0.0053	0.8689	0.0048	0.8835	0.0068
182	GLY	0.8322	0.0022	0.8215	0.0027	0.8267	0.0029
183	ARG	0.8633	0.0065	0.9040	0.0094	0.9328	0.0048
184	GLU	0.8335	0.0019	0.8272	0.0022	0.8292	0.0021
185	GLU	0.8389	0.0022	0.8335	0.0026	0.8305	0.0035
186	ARG	0.7909	0.0053	0.8206	0.0135	0.8393	0.0121
187	GLN	0.8524	0.0012	0.8350	0.0025	0.8420	0.0029
189	VAL	0.8175	0.0015	0.8057	0.0025	0.8100	0.0028
190	PHE	0.8288	0.0058	0.8240	0.0060	0.8889	0.0104
192	PHE	0.6494	0.0018	0.6477	0.0027	0.6578	0.0029
193	GLU	0.8424	0.0033	0.7966	0.0040	0.8535	0.0035
194	SER	0.8491	0.0038	0.8356	0.0035	0.8517	0.0064
195	GLY	0.8201	0.0022	0.8264	0.0029	0.8322	0.0032
198	PHE	0.8460	0.0021	0.8439	0.0018	0.8377	0.0024
199	LYS	0.8398	0.0008	0.8329	0.0029	0.8561	0.0025
200	ILE	0.8382	0.0013	0.8394	0.0020	0.8440	0.0016
201	GLN	0.8278	0.0010	0.8161	0.0021	0.8445	0.0035
202	VAL	0.8128	0.0012	0.8089	0.0030	0.8263	0.0030
203	LEU	0.8656	0.0027	0.8652	0.0036	0.8669	0.0032
204	VAL	0.8668	0.0007	0.8649	0.0033	0.8768	0.0036
205	GLU	0.8658	0.0025	0.8792	0.0029	0.8670	0.0035
207	ASP	0.8557	0.0047	0.8822	0.0092	0.8842	0.0110
208	HIS	0.8871	0.0027	0.8809	0.0030	0.9029	0.0033

209	PHE	0.8476	0.0008	0.8421	0.0024	0.8532	0.0045
210	LYS	0.8415	0.0020	0.8373	0.0032	0.8405	0.0033
212	ALA	0.8824	0.0016	0.8750	0.0022	0.8830	0.0017
213	VAL	0.8416	0.0024	0.8591	0.0027	0.8473	0.0025
214	ASN	0.8857	0.0019	0.8795	0.0021	0.8864	0.0023
215	ASP	0.8688	0.0020	0.8676	0.0029	0.8700	0.0033
216	ALA	0.8262	0.0014	0.8120	0.0018	0.8155	0.0020
218	LEU	0.8887	0.0015	0.8650	0.0061	0.8794	0.0033
219	LEU	0.8723	0.0006	0.8697	0.0032	0.8817	0.0040
220	GLN	0.8476	0.0021	0.8647	0.0036	0.8611	0.0035
221	TYR	0.8407	0.0020	0.8568	0.0030	0.8381	0.0046
222	ASN	0.8605	0.0016	0.8577	0.0026	0.8646	0.0036
223	HIS	0.8476	0.0016	0.8567	0.0027	0.8502	0.0015
224	ARG	0.8252	0.0027	0.8344	0.0036	0.8478	0.0060
225	VAL	0.7168	0.0018	0.7358	0.0033	0.7315	0.0039
227	LYS	0.7843	0.0060	0.8119	0.0069	0.7746	0.0127
228	LEU	0.8382	0.0015	0.8293	0.0022	0.8277	0.0024
229	ASN	0.8621	0.0021	0.8757	0.0033	0.8906	0.0029
230	GLU	0.8634	0.0017	0.8652	0.0023	0.8677	0.0028
231	ILE	0.8608	0.0020	0.8612	0.0020	0.8587	0.0024
232	SER	0.7517	0.0017	0.7564	0.0021	0.7545	0.0021
233	LYS	0.9015	0.0021	0.8963	0.0024	0.9153	0.0035
234	LEU	0.8460	0.0014	0.8553	0.0018	0.8428	0.0027
235	GLY	0.8540	0.0022	0.8448	0.0015	0.8501	0.0021
236	ILE	0.8411	0.0015	0.8400	0.0025	0.8477	0.0028
237	SER	0.8355	0.0015	0.8645	0.0021	0.8263	0.0045
238	GLY	0.8659	0.0018	0.8612	0.0032	0.8661	0.0039
239	ASP	0.8245	0.0022	0.8298	0.0026	0.8233	0.0029
240	ILE	0.8550	0.0019	0.8591	0.0019	0.8522	0.0014
241	ASP	0.8645	0.0025	0.8540	0.0026	0.8504	0.0021
242	LEU	0.8160	0.0019	0.8073	0.0021	0.8077	0.0024
243	THR	0.8766	0.0024	0.8559	0.0034	0.8721	0.0033
244	SER	0.8503	0.0013	0.8363	0.0022	0.8473	0.0020
245	ALA	0.8513	0.0021	0.8607	0.0022	0.8605	0.0020
246	SER	0.8431	0.0017	0.8365	0.0019	0.8399	0.0021
247	TYR	0.8717	0.0015	0.8374	0.0018	0.8545	0.0018
248	THR	0.8505	0.0017	0.8581	0.0018	0.8646	0.0019
249	MET	0.7620	0.0024	0.7573	0.0043	0.7881	0.0040
250	ILE	0.8232	0.0015	0.8148	0.0021	0.8048	0.0015

Table S3. Methyl-axis side-chain order parameters (O^2) with 1 SD errors for the three galectin-3C complexes.

Residue	Type	Meta		Para		Ortho	
		O^2	SD	O^2	SD	O^2	SD
1141	LEU	0.489	0.001	0.492	0.001	0.56	0.053
1142	LEU	0.461	0	0.464	0	0.468	0
1151	ILE	0.3	0.012	0.309	0.007	0.32	0.048
1152	ILE	0.605	0.058	0.629	0.016	0.586	0.069
1161	VAL	0.408	0.001	0.765	0.099	0.775	0.046
1202	LEU	0.705	0.002	0.538	0.022	0.723	0.003
1221	LEU	0.664	0.002	0.681	0.002	0.73	0.012
1261	VAL	0.704	0.003	0.856	0.042	0.706	0.003
1272	VAL	0.489	0.021	0.375	0.107	0.26	0.001
1301	MET	0.467	0	0.462	0.017	0.474	0
1311	LEU	0.648	0.003	0.661	0.004	0.622	0.004
1312	LEU	0.587	0.001	0.643	0.001	0.642	0.001
1321	ILE	0.337	0	0.349	0	0.334	0
1322	ILE	0.8	0.001	0.829	0.001	0.823	0.001
1331	THR	0.912	0.002	0.921	0.001	0.962	0.002
1341	ILE	0.554	0.038	0.527	0.033	0.491	0.032
1342	ILE	0.833	0.001	0.888	0.011	0.778	0
1351	LEU	0.431	0.055	0.374	0.001	0.364	0.001
1352	LEU	0.383	0.003	0.406	0.005	0.381	0.021
1371	THR	0.751	0.013	0.738	0.002	0.669	0.017
1381	VAL	0.873	0.001	0.901	0.001	0.921	0.002
1421	ALA	0.857	0.002	0.917	0.003	0.88	0.001
1451	ILE	0.898	0.006	0.826	0.069	0.758	0.001
1452	ILE	0.674	0	0.759	0.001	0.715	0.001
1461	ALA	0.912	0.002	0.934	0.001	0.603	0.04
1471	LEU	0.38	0.082	0.403	0.13	0.394	0.035
1472	LEU	0.407	0.212	0.257	0.001	0.382	0.187
1551	VAL	0.897	0.004	0.851	0.002	0.773	0.006
1552	VAL	0.825	0.001	0.846	0.001	0.851	0.001
1561	ALA	0.934	0.004	0.939	0.007	0.858	0.009
1701	VAL	0.394	0.002	0.579	0.065	0.678	0.06
1702	VAL	0.496	0.002	0.435	0.002	0.444	0.002
1711	ILE	0.383	0.001	0.439	0.023	0.307	0.001
1712	ILE	0.844	0.001	0.863	0.001	0.815	0.001
1721	VAL	0.858	0.001	0.875	0.001	0.886	0.001
1722	VAL	0.819	0.003	0.837	0.002	0.813	0.002
1771	LEU	0.351	0.001	0.363	0.001	0.361	0

1772	LEU	0.4	0.001	0.453	0.001	0.422	0.001
1891	VAL	0.864	0.008	0.775	0.046	0.644	0.001
1892	VAL	0.664	0.001	0.777	0.013	0.796	0.04
2001	ILE	0.664	0	0.773	0.085	0.722	0.001
2002	ILE	0.77	0.001	0.798	0.001	0.811	0.001
2021	VAL	0.793	0.001	0.887	0.001	0.752	0.001
2031	LEU	0.575	0.001	0.595	0.001	0.596	0
2032	LEU	0.634	0.001	0.592	0.001	0.605	0.001
2041	VAL	0.898	0.001	0.928	0.001	0.901	0.001
2042	VAL	0.882	0.001	0.905	0.001	0.86	0.001
2111	VAL	0.843	0.001	0.828	0.001	0.866	0.001
2112	VAL	0.869	0.001	0.964	0.001	0.868	0.003
2121	ALA	0.91	0.001	0.932	0.001	0.941	0.001
2131	VAL	0.856	0.002	0.828	0.003	0.866	0.002
2132	VAL	0.541	0.001	0.572	0.001	0.566	0.001
2161	ALA	0.882	0	0.874	0	0.877	0.001
2181	LEU	0.791	0.004	0.778	0.003	0.864	0.004
2182	LEU	0.656	0.007	0.696	0.003	0.652	0.002
2191	LEU	0.876	0.004	0.749	0.003	0.753	0.003
2251	VAL	0.758	0.001	0.778	0.001	0.744	0.002
2252	VAL	0.723	0.001	0.87	0.038	0.729	0.001
2281	LEU	0.624	0.002	0.624	0.002	0.614	0.002
2282	LEU	0.636	0.003	0.636	0.002	0.622	0.002
2311	ILE	0.644	0.001	0.609	0.001	0.649	0.001
2312	ILE	0.863	0.001	0.88	0.001	0.801	0.121
2341	LEU	0.789	0.012	0.698	0.002	0.679	0.001
2342	LEU	0.594	0.002	0.681	0.002	0.672	0.003
2361	ILE	0.594	0.032	0.589	0.057	0.528	0.001
2362	ILE	0.869	0.001	0.866	0.001	0.855	0.001
2401	ILE	0.472	0.001	0.534	0	0.519	0
2431	THR	0.764	0.033	0.669	0.002	0.757	0.032
2451	ALA	0.855	0.005	0.665	0.003	0.971	0.004
2481	THR	0.812	0.003	0.613	0.001	0.758	0.002
2491	MET	0.47	0	0.512	0.055	0.469	0
2501	ILE	0.455	0.001	0.427	0.049	0.384	0
2502	ILE	0.691	0.002	0.65	0.015	0.697	0.03

[a] The first three numbers indicate the residue number, while the forth number indicates the stereochemistry with '1' representing single methyl groups (in Ala, Thr, and Met) as well as methyl groups Cγ1 (Val), Cδ1 (Leu) and Cδ1 (Ile), and '2' representing methyl groups Cγ2 (Val), Cδ2 (Leu) and Cγ2 (Ile).

Table S4. Difference in conformational entropy between the bound and free states for M–, P– and O–galectin-3C, obtained from the MD simulations.[a]

Complex	$-T\Delta S_{conf}$(Prot)	$-T\Delta S_{conf}$(Lig)	$-T\Delta S_{conf}$(tot)	$-T\Delta\Delta S_{conf}$(Prot) [b]	$-T\Delta\Delta S_{conf}$(Lig) [b]
M–galectin-3C	9 ± 3	19.6 ± 0.4	29 ± 3	–5.0 ± 3.7	1.4 ± 0.5
P–galectin-3C	14 ± 3	20.4 ± 0.3	34 ± 3	2.5 ± 3.7	2.6 ± 0.4
O–galectin-3C	14 ± 3	16.1 ± 0.4	30 ± 3	2.5 ± 3.7	–3.9 ± 0.5

[a] $-T\Delta S_{conf}$ is the entropy difference upon ligand binding, based on all dihedral angles, divided into contributions from the protein and the ligand. $-T\Delta S_{bb}$ is based on the backbone ϕ and ψ angles only. Units are kJ/mol. Errors are given as ±1 SD.

[b] Inter-complex comparison.

Table S5. Conformational entropy and solvation free energy of the free ligands.[a]

Ligand	$-T\Delta S_{conf}$ [a]	$-T\Delta S_{rot}$ [b]	$-T\Delta S_{trans}$ [b]	$-T\Delta S_{water}$ [b]	ΔG_{solv} [c]
M	105.3 ± 0.1	-0.41 ± 0.02	-9.1 ± 0.2	-9.5 ± 0.2	–183.6 ± 1.4
P	106.1 ± 0.1	-0.50 ± 0.01	-9.6 ± 0.1	-10.1 ± 0.1	–185.6 ± 1.2
O	105.2 ± 0.1	-0.42 ± 0.01	-9.0 ± 0.1	-9.4 ± 0.1	–183.2 ± 1.6

[a] determined by MD simulations (see the main text for methods) and referenced to the entropy of a free rotor. Errors are given as ±1 SD. [b] determined by GIST calculations (see the main text for methods). [c] determined by COSMO-RS calculations (see the main text for methods). Units are kJ/mol. Errors are given as ±1 SD.

Table S6. Results of the MM/GBSA and interaction entropy calculations.[a]

Ligand	ΔE_{vdW}	ΔE_{el}	ΔG_{GB}	ΔG_{np}	$\Delta E_{el} + \Delta G_{GB}$	ΔG_{tot}	$-T\Delta S$	ΔG_{bind}
M	-112.3 ± 0.4	-272 ± 4	263 ± 3	-19.1 ± 0.1	-9 ± 1	-141 ± 1	143 ± 6	2 ± 6
P	-113.1 ± 0.3	-295 ± 4	276 ± 3	-19.4 ± 0.1	-19 ± 1	-152 ± 1	138 ± 9	-14 ± 9
O	-116.8 ± 0.6	-234 ± 5	242 ± 3	-19.1 ± 0.1	8 ± 1	-128 ± 1	132 ± 6	4 ± 6

[a] The terms are the van der Waals energy (ΔE_{vdW}), the electrostatic energy (ΔE_{el}), the generalized Born solvation free energy (ΔG_{GB}), the non-polar solvation free energy (ΔG_{np}) and the interaction entropy ($-T\Delta S$). $\Delta G_{tot} = \Delta E_{vdW} + \Delta E_{el} + \Delta G_{GB} + \Delta G_{np}$ and $\Delta G_{bind} = \Delta G_{tot} - T\Delta S$. Units are kJ/mol. Errors are given as ±1 SEM.

MM/GBSA and interaction entropy calculations

We performed MM/GBSA calculations[1–3] for the three complexes between M, P and O and galectin-3C. The calculations were based on the 10 × 100 ns unrestrained MD simulations of the three complexes, using the mmpbsa.py utility of AMBER.[4] The calculations employed the latest generalized Born method GB-Neck2 (igb = 8) with modified Bondii radii (mbondi3),[5] and a dielectric constant of 80 outside the solute and 1 inside the solute. The non-polar solvation free energy was calculated from the solvent accessible surface, using $\Delta G_{np} = \alpha$ SASA + b, with α = 0.0227 kJ/mol/Å^2 and b = 3.85 kJ/mol.[6] The calculations employed the single-trajectory approach (i.e. structures for the free protein and the free ligand were extracted from the simulation of the complex).[2] Snapshots were sampled every 10 ps, giving 100 000 in total. The uncertainty was calculated from the standard deviation over 200 batches of 5 ns simulations divided by $\sqrt{200}$.

Entropies for the binding of the three ligands to galectin-3C were calculated by the interaction entropy (IE) approach,[7] using the MM/GBSA data, but the sampling was increased to every 10 fs, as in the original IE article,[7] and energies were sampled for 10 ns. Results were obtained for 10 independent simulations and the uncertainty was estimated from the standard deviation over these 10 simulations divided by $\sqrt{10}$.

The MM/GBSA and IE results are collected in Table S6. It can be seen that the binding affinities are dominated by the electrostatic and GB solvation terms. However, these two terms are closely anticorrelated and nearly cancel (their sums are –10, –19 and 8 kJ/mol for M, P and O, respectively). Therefore, the net binding is instead dominated by the van der Waals term, –112 to –117 kJ/mol. The calculated binding free energy is least favorable for O, –128 ± 1 kJ/mol without and 4 ± 6 kJ/mol with the IE entropies, in accordance with experiments, but that of P (–152 ± 1 or –14 ± 9

kJ/mol) is appreciably lower than that of M (–141 ± 1 or 2 ± 6 kJ/mol). Still, the correlation coefficient to the experimental results is reasonable, R = 0.43, and similar to what is reported in other studies.[2,3] However, the absolute values and the differences are strongly overestimated, which is often observed for MM/GBSA.[2] Therefore, we refrain from any deeper discussion of the cause of the differences between the three ligands, except that they arise mainly from the electrostatics and solvation terms, whereas differences in the van der Waals and entropy terms are rather small (up to 4 and 5 kJ/mol, respectively). The interaction entropies are very sensitive to details of the calculations, as will be discussed in a separate publication. However, P always gives a lower entropy than the other two ligands by 1–5 kJ/mol.

Table S7. Residues included in each shell in Figure 9 of the main text.

Distance (Å)	Residues
0–2:	158, 162, 174, 184
2–3:	144, 146, 160, 172, 181, 186, 237, 238
3–4:	145, 165
4–5:	182, 239
5–6:	148, 173, 175, 176
6–7:	116, 143, 147, 155, 159, 170, 183, 185, 236, 240
7–8:	163, 171, 180, 221, 224
8–9:	118, 139, 142, 150, 157, 177, 179, 235
9–11:	138, 154, 156, 164, 168, 187, 190, 192, 198, 200, 241, 242
11–13:	115, 119, 134, 136, 137, 167, 169, 178, 194, 219, 225, 233, 234
13–15:	120, 135, 151, 189, 193, 195, 202, 209, 213, 218, 220, 222, 223, 231, 243, 245
15–17:	122, 132, 133, 199, 201, 208, 212, 214, 244
17–19:	126, 203, 204, 207, 210, 227, 228, 230, 232, 246
19–21:	125, 130, 131, 205, 215, 216, 247
21–23:	127, 229, 248
23–25	129
25–26:	249, 250

References

(1) Kollman, P. A.; Massova, I.; Reyes, C. M.; Kuhn, B.; Huo, S.; Chong, L.; Lee, M. C.; Lee, T.; Duan, Y.; Wang, W.; et al. Calculating Structures and Free Energies of Complex Molecules: Combining Molecular Mechanics and Continuum Models. *Acc. Chem. Res.* **2000**, *33*, 889–897.

(2) Genheden, S.; Ryde, U. The MM/PBSA and MM/GBSA Methods to Estimate Ligand-Binding Affinities. *Expert Opin. Drug Discov.* **2015**, *10*, 449–461.

(3) Wang, E.; Sun, H.; Wang, J.; Wang, Z.; Liu, H.; Zhang, J. Z. H.; Hou, T. End-Point Binding Free Energy Calculation with MM/PBSA and MM/GBSA: Strategies and Applications in Drug Design. *Chem. Rev.* **2019**, *119*, 9478–9508.

(4) Case, D. A.; Berryman, J. T.; Betz, R. M.; Cerutti, D. S.; Cheatham III, T. E.; Darden, T. A.; Duke, R. E.; Giese, T. J.; Gohlke, H.; Goetz, A. W.; et al. AMBER 2015. *University of California, San Francisco*. 2015.

(5) Nguyen, H.; Roe, D. R.; Simmerling, C. Improved Generalized Born Solvent Model Parameters for Protein Simulations. *J. Chem. Theory Comput.* **2013**, *9*, 2020–2034.

(6) Kuhn, B.; Kollman, P. A. Binding of a Diverse Set of Ligands to Avidin and Streptavidin: An Accurate Quantitative Prediction of Their Relative Affinities by a Combination of Molecular Mechanics and Continuum Solvent Models. *J. Med. Chem.* **2000**, *43*, 3786–3791.

(7) Duan, L.; Liu, X.; Zhang, J. Z. H. Interaction Entropy: A New Paradigm for Highly Efficient and Reliable Computation of Protein–Ligand Binding Free Energy. *J. Am. Chem. Soc.* **2016**, *138*, 5722–5728.
